# Amyloid Plaques Ameliorate Memory Deficits and Hippocampal Neuron Loss in an Aβ4-42-Driven Alzheimer’s Disease Mouse Model

**DOI:** 10.1007/s12035-026-05912-x

**Published:** 2026-05-15

**Authors:** Silvia Zampar, Merle Fricke, Florian Kremser, Ivan Talucci, Hans Michael Maric, Hans-Wolfgang Klafki, Agueda Rostagno, Olaf Jahn, Sascha Weggen, Jorge Ghiso, Oliver Wirths

**Affiliations:** 1 Department of Psychiatry and Psychotherapy, University Medical Center (UMG), Georg-August-University, Von-Siebold-Str. 5, 37075 Göttingen, Germany; 2 Rudolf Virchow Center for Integrative and Translational Bioimaging, University of Würzburg, Würzburg, Germany; 3 Department of Neurology, University Hospital Würzburg, Würzburg, Germany; 4 Neuroproteomics Group, Department of Molecular Neurobiology, Max Planck Institute for Multidisciplinary Sciences (City Campus), Göttingen, Germany; 5 Department of Neuropathology, Heinrich-Heine-University, Düsseldorf, Germany; 6 Department of Pathology, New York University School of Medicine, New York, NY USA

**Keywords:** Alzheimer’s disease, Abeta, Mouse model, N-terminal truncation, Behavior, Neuron loss, Amyloid pathology

## Abstract

Extracellular deposition of amyloid-β (Aβ) peptides in the form of plaques is the most prominent pathological hallmark of Alzheimer’s disease (AD). The postulated central pathophysiological role of fibrillary Aβ plaques has, however, been questioned, and small, soluble, pre-fibrillar Aβ aggregates (oligomers) have been implicated as the crucial neurotoxic species in AD etiology. While the relationship between insoluble amyloid plaques and soluble Aβ oligomers remains unclear, it has been hypothesized that plaques may serve as reservoirs, sequestering toxic Aβ oligomers in the initial stages of the disease. Next to the canonical “full-length” Aβ1-40 and Aβ1-42 peptides, a variety of N-terminally truncated Aβ variants are present in AD brain tissue, with Aβ4-42 peptides showing high abundance. The detrimental effects of these N-terminally truncated peptides have been previously studied using the Tg4-42^hom^ mouse line, which displays neuron loss and cognitive deficits and accumulates Aβ4-42 peptides in the CA1 region of the hippocampus albeit without amyloid plaque formation. This study aimed to investigate the relationship between soluble Aβ4-42 peptides and insoluble extracellular Aβ deposits by crossing the Tg4-42^hom^ line with the plaque-bearing 5XFAD mouse model. We found that extracellular amyloid deposits in the hippocampus did not aggravate spatial memory deficits in Tg4-42^hom^ mice but rescued recognition memory deficits. Moreover, while proximal CA1 pyramidal neuron loss in the hippocampus of Tg4-42^hom^ mice was not affected by crossing with the 5XFAD line, a reduced loss of distal pyramidal neurons was observed in the filial line. Biochemically, 5XFAD/Tg4-42^hom^ mice showed a trend towards increased levels of insoluble Aβ4-x peptides in the hippocampus. Taken together, these findings support the importance of soluble Aβ oligomers in the pathogenesis of AD and provide evidence for the hypothesis that amyloid plaques provide buffering capacity.

**Supplementary Information:**

The online version contains supplementary material available at 10.1007/s12035-026-05912-x.

## Introduction

The most prominent pathological hallmark of Alzheimer’s disease (AD) is the cerebral deposition of amyloid-β (Aβ) peptides in the form of extracellular amyloid plaques. The postulated central role of fibrillar Aβ plaques in the pathogenesis, as proposed by the classical amyloid cascade model, has been questioned, as the amount and localization of these deposits do not correlate with the specific disease states [1]. With regard to amyloid-β peptide deposition, approximately 40% of non-demented individuals aged > 50 met criteria for a neuropathological diagnosis of AD to some degree [2]. Additionally, clinical studies reported individuals who can accumulate loads of amyloid-β plaques and tangles equivalent to demented AD cases without experiencing dementia [3, 4]. These observations have spurred an adjustment of the amyloid hypothesis, emphasizing soluble, pre-fibrillar Aβ oligomers rather than insoluble fibrillar extracellular Aβ deposits as the crucial aggregate species implicated in AD etiology [5–8]. Soluble oligomeric forms of Aβ have been described in post mortem tissues of AD patients [7, 9, 10] and have been found to better correlate with the risk and severity of the disease than insoluble amyloid plaques [10, 11]. Further supporting the Aβ oligomer hypothesis, the antibody Lecanemab, FDA-approved in 2023 as a disease-modifying treatment for AD [12], was raised against soluble oligomers/protofibrils [13].

Several groups have addressed the still-debated relationship between insoluble amyloid plaques and soluble Aβ oligomers. It has been hypothesized that plaques may serve as reservoirs, sequestering toxic Aβ oligomers, thus preventing their toxicity at the initial stages of the pathology. With the progression of the disease and saturation of the reservoirs, plaques might start to release the soluble Aβ aggregates (or fail to sequester them), now free to bind to other targets and to exert their detrimental effects [14, 15]. The findings of Koffie and colleagues, who analyzed synaptic loss as a function of distance from amyloid plaques with array tomography, support the idea of amyloid plaques acting as potential reservoirs of toxic soluble Aβ species. In both AD transgenic mice and human AD brains, synaptic density decreased concentrically around plaques, returning close to normal values beyond 50 μm of distance [16, 17]. Esparza and co-workers further tested this hypothesis by measuring the Aβ binding capacity of amyloid plaques in human unfixed frozen brain sections. Amyloid plaques were able to bind synthetic Aβ1–42 peptides, though comparably between AD patients and high-pathology controls [18].

Next to the canonical “full-length” Aβ_1–40_ and Aβ_1–42_ peptides, a variety of N-terminally truncated Aβ variants starting at positions 2, 3, pE3, 4, 5, 8 or 9 have been detected in AD brain tissue [19, 20]. Among the different N-truncated isoforms, AβpE3-42 and Aβ4–42 were detected with high abundance, at comparable or even exceeding levels compared to Aβ1–42 [21–23]. Aβ variants starting with phenylalanine at position 4 represent the major N-truncated Aβ species in parenchymal Aβ plaques detected by classical Edman protein sequencing [24] and are also present within blood vessels in AD cases [23, 25, 26].

A mouse model (Tg4-42^hom^) exclusively expressing Aβ4–42 peptides under the control of the murine neuron-specific Thy1 promoter has been generated. Intriguingly, these mice do not develop any amyloid plaque pathology, but exhibit abundant hippocampal neuron loss, together with learning and memory deficits in an age- and gene-dose-dependent manner [27, 28].

The relationship between insoluble amyloid plaques and soluble Aβ oligomers is not fully understood. In this study, we investigated the interrelation between soluble, neurotoxic Aβ4–42 peptides and insoluble extracellular Aβ deposits by crossing the plaque-bearing 5XFAD mouse model with the Tg4-42^hom^ mouse line exclusively expressing N-terminally truncated Aβ4–42 peptides.

## Materials and Methods

### Experimental Animals

The Tg4-42^hom^ transgenic mouse model of AD overexpresses a genetic construct comprising the human Aβ_4–42_ sequence fused to the murine thyrotropin-releasing hormone (TRH) signal peptide under the control of the Thy1 promoter [27]. Tg4-42^hom^ mice were generated and maintained in a homozygous manner on a C57BL/6 J genetic background. Heterozygous 5XFAD mice (line Tg6799) [29] overexpress the human APP695 (carrying the Swedish, Florida and London mutations), as well as the mutant human presenilin-1 (PSEN-1), (with the M146L and L286V mutations), under the control of the murine Thy1 promoter. The 5XFAD line was backcrossed for more than ten generations to C57BL/6 J wild type mice and was maintained on a C57BL/6 J genetic background. 5XFAD and Tg4-42^hom^ mice were crossed to generate the 5XFAD/Tg4-42^hom^ line, expressing human APP and PSEN1 in a heterozygous and the Tg4-42 transgene in a homozygous fashion. C57BL/6 J mice (WT) served as controls (Jackson Laboratories, Bar Harbor, ME, USA). In this study, only female animals were used. Animals were kept on a 12 h/12 h inverted light/dark cycle (light phase from 8 PM to 8 AM) and were provided with food and water ad libitum. All animals were handled according to German guidelines for animal care and all experiments were approved by the local animal care and use committee (Lower Saxony State Office for Consumer Protection and Food Safety, 17/2512).

### Novel Object Recognition

To test for recognition memory, the novel object recognition (NOR) task was performed [30]. To habituate the animals to the testing arena (50 × 50 cm), each mouse was placed in the apparatus 24 h prior to the first training day, and left to explore freely for 5 min. On the training trial, two identical objects were positioned in the arena. During the test trial on the following day, one of the two familiar identical objects was substituted with a new object, different in shape, color, and texture. The mice could freely explore the objects on both trials and the exploration time for each object was manually recorded whenever the animal sniffed the objects while looking at them. Object preferences were reported as % of explored time to total exploration time. The recognition performances were quantified using the Discrimination Index (DI), measured as follows:

$$\mathrm{D}\mathrm{I}=\frac{\mathrm{T}\mathrm{I}\mathrm{M}\mathrm{E}\left(\mathrm{n}\mathrm{o}\mathrm{v}\mathrm{e}\mathrm{l}\right)-\mathrm{T}\mathrm{I}\mathrm{M}\mathrm{E}\left(\mathrm{f}\mathrm{a}\mathrm{m}\mathrm{i}\mathrm{l}\mathrm{i}\mathrm{a}\mathrm{r}\right)}{\mathrm{T}\mathrm{I}\mathrm{M}\mathrm{E}(\mathrm{n}\mathrm{o}\mathrm{v}\mathrm{e}\mathrm{l}+\mathrm{f}\mathrm{a}\mathrm{m}\mathrm{i}\mathrm{l}\mathrm{i}\mathrm{a}\mathrm{r})}$$

A DI equivalent to zero indicates an equal exploration of the novel and familiar objects, while a positive or a negative DI represents a preference for the novel or familiar object, respectively.

### Morris Water Maze

The Morris Water Maze test (MWM) was used to assess spatial reference memory [31]. The apparatus consisted of a circular pool (ø 110 cm) filled with water made opaque with non-toxic white paint and containing a submerged platform (ø 10 cm). At first, animals performed a “cued training” in which the submerged platform was marked with a triangular visible flag. Mice were placed in the pool and the latency to reach the flagged platform was recorded during three consecutive days (4 trials per day, each one for a maximum of 1 min). Twenty-four hours after the last cued training trial, a 5-day “acquisition training” was carried out. Proximal visual cues were added around the pool, the flag was removed from the platform and mice were trained to learn to localize the hidden platform in the four daily trials. Each session lasted a maximum of 60 s and ended once the mouse reached the hidden platform, whose location remained fixed in the target quadrant during the acquisition training. If a mouse failed to reach the platform, it was gently guided and placed on the platform for 10 s. Finally, a “probe trial” was used to assess spatial reference memory 24 h after the last acquisition training trial. The proximal and distal cues remained in the same position, but the platform was removed from the pool. A 10-min break occurred in between the trials, during which the mice were dried and kept under infrared light to prevent hypothermia. All trials were recorded using video-tracking software (ANY-maze, Stoelting Europe) and the escape latency, swimming speed, time into the platform/target quadrant, and entries into the platform/target quadrant were recorded.

### Tissue Collection and Preservation

Mice were deeply anesthetized with carbon dioxide and sacrificed via cervical dislocation. After decapitation, the brain was rapidly and carefully dissected on ice. The right hemispheres were positioned into embedding cassettes and post-fixed in a light-protected 4% phosphate-buffered formaldehyde solution (Roti®-Histofix 4%, Roth, Karlsruhe, Germany) at 4 °C for at least a week prior to paraffin embedding. The left hemisphere was further processed, and the hippocampus was dissected, quickly frozen on dry ice, and stored at −80 °C.

### Immunohistochemistry on Paraffin Sections

Sagittal 4 μm paraffin brain sections were deparaffinized in xylene and rehydrated by washes in decreasing ethanol concentrations. The slides were treated with 0.3% H_2_O_2_ in 0.01 M PBS for 30 min to block endogenous peroxidases and antigen retrieval was performed by boiling sections in 0.01 M citrate buffer pH 6.0. Membrane permeabilization was achieved by a 15 min incubation in 0.01 M PBS incl. 0.1% Triton X-100. Non-specific binding sites were blocked with incubation in milk and fetal calf serum in PBS for 1 h prior to the addition of the primary antibodies. The following primary antibodies were used: 82E1 (mouse, #JP10323, IBL International, Hamburg, Germany); 18H6 (mouse [23]), IBA1 (guinea pig, #234308, RRID:AB_2924932, Synaptic Systems, Göttingen, Germany), D3E10 (rabbit, #12843, RRID:AB_2798041, Cell Signaling Technology, Danvers, USA), GFAP (rabbit, #100139-RP02, Sino Biological Europe, Eschborn, Germany). The primary antibodies were diluted in 10% fetal calf serum in 0.01 M PBS and incubated overnight at RT. Biotinylated goat-anti-mouse antibodies (#115-065-044; RRID: AB_2338561 (Jackson Immuno Research, Ely, UK)) were applied for 1.5 h in a humid chamber at 37 °C and visualized using the ABC method with a Vectastain kit (Vector Laboratories, Burlingame, USA) and diaminobenzidine as chromogen. The fluorescent secondary antibodies (anti-guinea pig-DyLight-550, #SA5-10095; RRID:AB_2556675; anti-rabbit-AlexaFluor-594, #R37117; RRID:AB_2556545, Thermo Fisher Scientific, Waltham, USA) were applied for 1.5 h at 37 °C in a humid chamber and counterstaining achieved with 4′,6-diamidin-2-phenylindol (DAPI).

### Quantification of Immunoreactivity

Diaminobenzidine-stained sections were evaluated in a blinded fashion using a BX51 microscope (Olympus, Hamburg, Germany), equipped with a Moticam Pro 282 camera (Motic, Wetzlar, Germany). Fluorescent images were acquired in a large-scale format with a Nikon TiE microscope equipped with a motorized stage and a cooled DS-Qi2 camera (Nikon, Amsterdam, Netherlands) and analyzed with NIS Elements imaging software (Nikon, Amsterdam, Netherlands). Brain regions of interest were delineated and the percentage area covered by immunoreactive signal was quantified on binarized 8-bit black-and-white images using a fixed intensity threshold with the ImageJ software package (V1.52, NIH, Bethesda, MD, USA). Quantification was carried out on 3 sections per animal distancing at least 30 µm.

### Quantification of Aβ4-x Signal Within Plaques

Sagittal 4 µm mouse brain sections were stained for Aβx-42 (D3E10) and Aβ4-x (18H6) and fluorescent images containing hippocampal and cortical areas were acquired in a large-scale format. Analysis was performed using the ImageJ software package (V2.14.0). After delineation, regions of interest (ROIs) were created around individual plaques using D3E10 immunostaining as a reference. Relative areas covered by D3E10 and 18H6 staining within these ROIs were taken and the ratio between both areas was calculated (relative area covered by Aβ4-x/relative area covered by Aβx-42). This ratio was defined as the relative plaque area covered by Aβ4-x peptides.

For calculation of Aβ4-x immunoreactivity within the border plaque area, the ROI was scaled down by 5 pixels and a new ROI was created containing the area between the original and the downscaled ROI. This new ROI defined the border plaque area and the relative area covered by Aβ4-x within this area was calculated. Per animal, 3 sections were analyzed with 15 plaques per section for the area and 10 plaques per section for the border area analysis. For illustration, data is shown in the form of SuperPlots, demonstrating all measurements and data of biological replicates used for statistics [32].

### Quantification of Neuron Numbers

Sagittal 4 μm paraffin brain sections (bregma between 0.60 and 1.08) were processed and stained with hematoxylin as previously described [33]. Images from the CA1 pyramidal layer were captured with 400 × magnification using an Olympus BX-51 microscope equipped with a Moticam Pro 282 A camera (Motic, Wetzlar, Germany). CA1 neuronal nuclei (*n* = 6 per group, 3 sections per animal, at least 50 µm apart), distinguishable from other cellular populations by size and color intensity, were manually counted by a blinded experimenter using the cell-counting tool implemented in ImageJ (version 1.52).

### Cell Culture

The generation of HEK293 human embryonic kidney cell lines with doxycycline-inducible ADAMTS4 expression and stable overexpression of human APP695 has been described previously [34]. Cells were maintained in DMEM with 10% FCS (v/v), 1% sodium pyruvate (v/v), 100 U/ml penicillin/streptomycin. Blasticidin and hygromycin were added as selection antibiotics (all media components from Thermo Fisher Scientific).

### Peptide Microarray and Immunoassay

Mapping of the 18H6 binding epitope was performed as described previously [34, 35] at 0.95 µg/ml. The human APP sequence 649–722 (Aβ numbering-23–51) was displayed as a 15-mer overlapping-peptide library in a microarray format on cellulose discs (offset one amino acid).

### Immunoprecipitation and Mass Spectrometry

Antibodies mAb 6E10 (#39320, RRID:AB_662798, BioLegend, San Diego, USA) and mAb 18H6 [23] were covalently coupled to Dynabeads M-270 Epoxy (Thermo Fisher Scientific) according to the instructions of the manufacturer, omitting Tween-20 from all wash and storage buffers as previously described [34]. For immunoprecipitation, 250 µl of HEK293-APP695wt/ADAMTS4 cell culture supernatant after induction of ADAMTS4 expression was used and immunoprecipitated with 15 µl of functionalized Dynabeads M-270 carrying either 6E10 or 18H6. Elution of Aβ peptides and subsequent analysis by matrix-assisted laser desorption/ionization time-of-flight mass spectrometry (MALDI-TOF-MS) were carried out as described previously [34, 36].

### Biotinylation of Aβ-Specific Antibodies

Biotinylated mAb 82E1 (0.1 mg/ml) (#10326, RRID:AB_2341281) was obtained from IBL International while biotinylated capture antibody 18H6 was prepared following the MSD Biotin Conjugation Quick Guide. Fifty microliters of a stock solution of mAb 18H6 (1 mg/ml) [23] were buffer-exchanged into MSD conjugation buffer (PBS, pH 7.9, preservative-free) on a Zeba Spin desalting column 40 K MWCO, 0.5 ml (Thermo Fisher Scientific). For biotinylation, a 0.9 nmol/μl Sulfo-NHS-LC biotin solution (EZ-link^(^™^)^ Micro Sulfo-NHS-LC-biotinylation kit, Thermo Fisher Scientific) was added to approximately 50 μg of IgG to have a challenge ratio of 10:1 and mixed immediately by vortexing. After 2 h incubation at 23 °C, excess biotin was removed by buffer exchange into MSD conjugate storage buffer (PBS, pH 7.4 containing 0.05% sodium azide) on a Zeba Spin desalting column (40 K MWCO, 0.5 ml). Glycerol was added to a final concentration of 50% (v/v) and the biotinylated antibodies were stored at −20 °C until use.

### Electrochemiluminescence Aβ Assays

Electrochemiluminescent immunoassays (Meso Scale Discovery (MSD), Rockville, USA) were developed to determine Aβ1-x and Aβ4-x levels in SDS-soluble brain homogenates. As capture antibodies, biotinylated 82E1 (IBL International; 1 µg/ml) or 18H6 ([23]; 0.6 µg/ml), as well as sulfo-tagged 4G8 (1:50, MSD) were used as detection antibodies on MSD GOLD 96-well Small Spot Streptavidin Plates (MSD) following the manufacturer’s protocol for the Human Aβ Antibody Set (Cat. No: F218H). Plates were coated with 25 μl of diluted biotinylated capture antibody in each well (1 µg/ml). After 1 h incubation at RT with shaking (700 rpm), the plate was washed 3 times with phosphate-buffered saline (PBS) containing 0.05% Tween 20 (PBS-T) and blocked with Diluent-35 (MSD). The plate was then washed again 3 times with PBS-T and incubated with 25 μl of detection antibody and 25 μl of sample per well for 2 h at RT with 700 rpm shaking. Finally, the plate was washed 3 times with PBS-T and read on an MSD QuickPlex SQ 120 reader after the addition of Gold Read Buffer (MSD). Standard curves were prepared using synthetic Aβ1–40 and Aβ4–40 (AnaSpec, Fremont, CA, USA) and data analysis, including standard curve calculations, was carried out with the Discovery Workbench 4.0.12 software package (MSD).

### Aβ Measurements in Mouse Brain Samples

For assay validation, SDS-soluble brain hemisphere extracts from 2-month-old, 5-month-old, and 10-month-old male 5XFAD mice were measured. In addition, deep-frozen hippocampi from 6-month-old 5XFAD and 5XFAD/Tg4-42^hom^ female mice were homogenized in 200 μl of Tris-buffered saline (TBS) buffer (120 mM NaCl, 50 mM Tris, pH 8.0 with complete protease inhibitor cocktail, Roche) per 100 mg tissue using a Bandelin Sonoplus sonicator (Sonotrode MS 1.5, 80% amplitude, 30 s with pulse). The homogenate was then centrifuged at 17,000 × g for 20 min at 4 °C and the supernatant containing TBS-soluble proteins was stored at −80 °C. 200 μl of 2% SDS were added to the pellet and the samples were further sonicated (80% amplitude, 20 s with pulse) on ice. The samples were centrifuged at 17,000 × g for 20 min at 4 °C, and the supernatant containing SDS-soluble proteins was transferred into a new tube, followed by addition of 1 μl of Benzonase (Merck Millipore) and an incubation for 10 min at RT prior to storage at −80 °C. To measure the levels of specific Aβ variants, electrochemiluminescent assays detecting Aβ_1-x_ and Aβ_4-x_ were performed according to the protocol for the Human Aβ Antibody Set of the manufacturer and were measured on an MSD QuickPlex SQ 120 (MSD, Rockville, USA) instrument.

### Quantitative Real-Time Polymerase Chain Reaction (qRT-PCR)

RNA was isolated from deep-frozen hippocampus samples at 6 months of age by homogenization in 1 ml of Trifast® reagent per 100 mg of sample and using a Dounce homogenizer (800 rpm) and processed as previously described [37]. DNAse digestion and reverse transcription of the purified RNA samples were carried out following the protocol of the manufacturer (Thermo Fisher Scientific). RT-PCR was performed with a Stratagene MX3000 Real-time Cycler with Blue S’Green qPCR Mix (Biozym, Hessisch Oldendorf, Germany), including ROX as an internal reference dye. Relative expression levels were calculated using the 2^−∆∆Ct^ method with normalization to the housekeeping gene β-actin [38]. The following primers were used:

Aβ4–42 forward: TCC GGC CAG AAC GTC GAT TC; Aβ4–42 reverse: GGA GAA GCA AGA CCT CTG C; human *APP* forward: TCT CGT TCC TGA CAA GTG CAA; human *APP* reverse: GCA AGT TGG TAC TCT TCT CAC TG; *ACTIN-β* forward: ATG GAG GGG AAT ACA GCC C; *ACTIN-β* reverse: TTC TTT GCA GCT CCT TCG TT; *CST7* forward: AAGCACTCCTGGGTTATTGG; *CST7* reverse: TGCCTAACTTCTGACACCCA; *TREM2* forward: CCCTCGAAACTCGATGACTC; *TREM2* reverse: TGCAGAAAGTACTGGTGGAGG; *APOE* forward: GAGCTGATCTGTCACCTCCG; *APOE* reverse: GGACTTGTTTCGGAAGGAGC; *GFAP* forward: CCTTCTGACACGGATTTGGT; *GFAP* reverse: ACATCGAGATCGCCACCTAC.

### Statistical Analyses

Data exploration was performed to assess the assumptions for parametric testing. In case of normal distribution, differences between groups were assessed with unpaired *t*-test, one-way analysis of variance (ANOVA) followed by Bonferroni multiple comparison or two-way ANOVA followed by Bonferroni multiple comparison correction. When the sample size was too small or assumptions for parametric tests were not met, differences between the groups were tested with Mann–Whitney test or Kruskal–Wallis test followed by Dunn’s multiple comparison test. All data were given as means ± standard deviation (SD). All statistics were performed using GraphPad Prism version 10.6 for Windows (GraphPad Software, San Diego, CA, USA).

## Results

### No Overt Differences in Amyloid Plaque Pathology Between 5XFAD and 5XFAD/Tg4-42^hom^ Mice

**Fig. 1:**
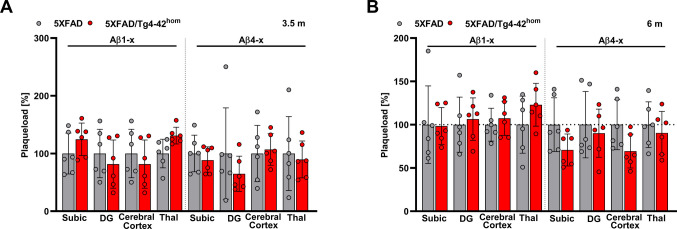
Aβ1-x and Aβ4-x immunoreactivity in 3.5- and 6-month-old 5XFAD and 5XFAD/Tg4-42^hom^ mice (*n* = 6). Sagittal paraffin sections from 3.5- (**A**) and 6-month-old mice (**B**) were stained with 82E1 (Aβ1-x) and 18H6 (Aβ4-x). Plaque load was quantified in the subiculum (Subic), dentate gyrus (DG), cerebral cortex and thalamus (Thal) and is represented with the 5XFAD group as a reference. All data are given as means ± SD. Unpaired *t-*test, *p < 0.05 (with adjustment for multiple testing)

Amyloid plaque pathology was analyzed and compared between 5XFAD and 5XFAD/Tg4-42^hom^ mice in the subiculum, dentate gyrus, cortex and thalamus at 3.5 and 6 months of age. No significant differences were observed with regard to the amount of Aβ1-x- or Aβ4-x-immunoreactive extracellular deposits in the analyzed brain regions (Fig. 1A, B**; **Suppl. Figure S1).

### Development of Sensitive Variant-Specific Aβ Peptide Immunoassays

**Fig. 2:**
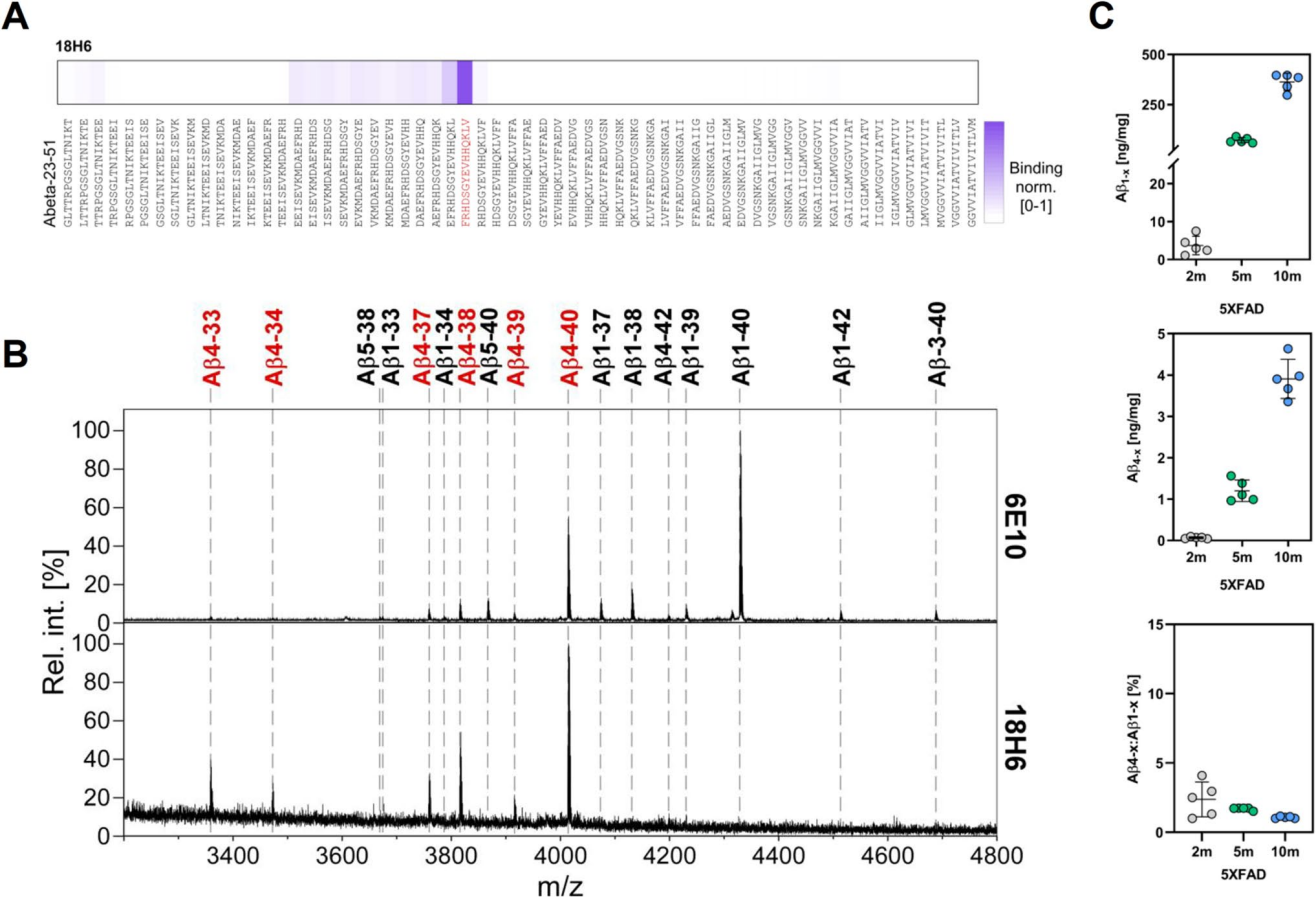
Peptide microarray screening of Aβ-23–51 against monoclonal antibody 18H6 confirmed selectivity for a peptide FRHDSGYEVHHQKLV corresponding to the N-terminus of Aβ4-x (**A**). IP-MS characterization of Aβ peptides immunoprecipitated with 6E10 or 18H6 from cell culture supernatants of HEK293 cells with stable co-expression of APP695 and ADAMTS4. With 18H6, only Aβ4-x peptide variants (in red) are detected (**B**). Quantification of Aβ1-x (**C**), Aβ4-x (**D**), and the Aβ4-x:Aβ1-x ratio (**E**) with electrochemiluminescence immunoassays in SDS extracts from brain hemispheres of 2-, 5-, and 10-month-old male 5XFAD mice (*n* = 5)

In order to quantify Aβ1-x and Aβ4-x peptide levels, selective immunoassays for measuring these Aβ variants were developed on the Meso Scale Discovery (MSD) technology platform employing 96-well small-spot streptavidin-pre-coated plates (see Supplementary Information). The strong preference of monoclonal antibodies 82E1 towards Aβ1-x and 18H6 towards Aβ4-x peptides has been previously demonstrated [23, 26, 39] and the binding epitope of 82E1 has recently been characterized in detail [35]. Within this study, we further characterized the binding epitope of the 18H6 antibody directed against Aβ4-x with peptide microarrays containing overlapping 15-mer Aβ peptides, at single amino acid resolution, ranging from position −23 to 51 as described previously [35]. This analysis revealed the strongest binding to FRHDSGYEVHHQKLV, a peptide corresponding to Aβ4-x starting with an N-terminal phenylalanine residue (Fig. 2A). We confirmed this selectivity by immunoprecipitation of conditioned media from HEK293 cells co-expressing APP695 and ADAMTS4, a protease known to generate Aβ4-x peptides [34, 40]. Immunoprecipitation with antibody 6E10 and subsequent MS analysis revealed the expected pattern of full-length as well as N-terminally truncated and elongated Aβ species, with Aβ1–40 and Aβ4–40 representing the most abundant variants [34]. In contrast, immunoprecipitation with 18H6 only detected peptides Aβ4–33, Aβ4–34, Aβ4–37, Aβ4–38, Aβ4–39, and Aβ4–40, underscoring its selectivity towards Aβ4-x peptide variants (Fig. 2B).

To establish quantitative sandwich immunoassays for Aβ4-x and Aβ1-x peptides, aliquots of antibodies 18H6 and 82E1 were conjugated with biotin to serve as capture antibodies. After blocking unspecific binding sites, 96-well small-spot streptavidin plates were coated with biotinylated mAb 18H6 or mAb 82E1 antibodies, and paired with SulfoTAG monoclonal antibody 4G8 (MSD) as a detection antibody. Subsequently, a series of validation experiments were carried out (Suppl. Figs. S2, S3; Suppl. Table S1). Finally, SDS-soluble extracts from brain hemispheres of 2-month-old, 5-month-old, and 10-month-old male 5XFAD mice were measured to assess the suitability of the assay with biological samples. Both Aβ1-x (Fig. 2C) and Aβ4-x (Fig. 2D) levels showed the expected age-dependent increase, with absolute levels of Aβ4-x peptides representing approximately 1–2% compared to Aβ1-x (Fig. 2E).

**Fig. 3:**
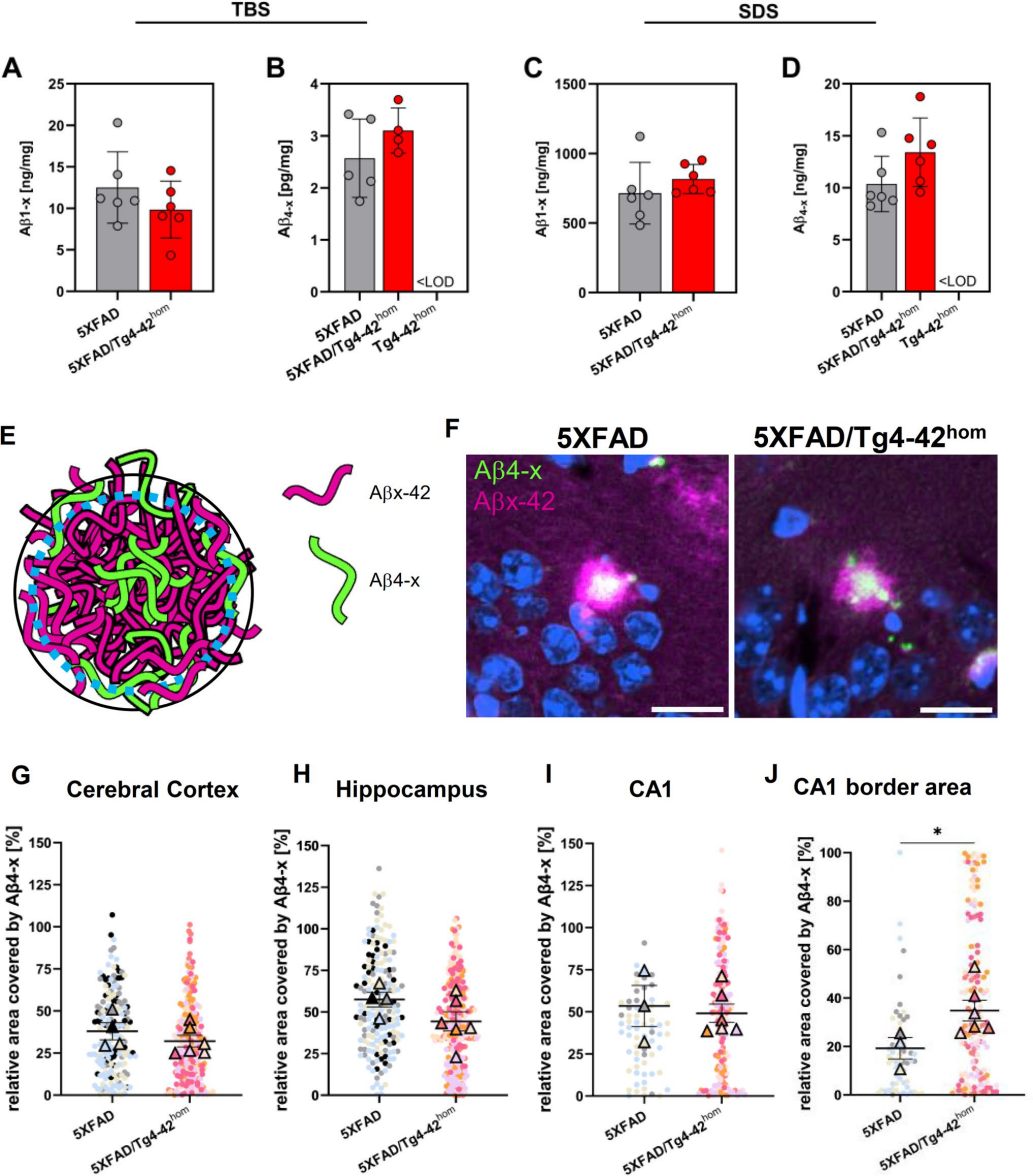
Electrochemiluminescence measurements of hippocampal Aβ peptide levels in 6-month-old 5XFAD/Tg4-42^hom^ mice and transgenic controls. Aβ1-x (**A**, **C**) and Aβ4-x (**B**, **D**) concentrations were measured with an electrochemiluminescence assay in TBS- (**A**, **B**) and SDS- (**C**, **D**) soluble hippocampal brain fractions of female 5XFAD and 5XFAD/Tg4-42^hom^ mice (*n* = 5–6). Schematic illustrating the quantification of Aβ4-x and Aβx-42 peptides (**E**). Examples of Aβ-positive plaques in in the CA1 region of 5XFAD and 5XFAD/Tg4-42.^hom^ mice (**F**). Quantification of Aβ4-x and Aβ42 immunoreactivity in extracellular deposits in the cortex (**G**), hippocampus (**H**), and CA1 area (**I**), as well as in the border area (depicted in **E**) of CA1 deposits (**J**). In G-I, data are shown in the form of SuperPlots, demonstrating all measurements and data of biological replicates used for statistics. All data are given as means ± SD. *p < 0.05 (unpaired *t*-test with Welch’s correction)

Given the changes in distal CA1 neuron numbers and in the behavioral performance of 6-month-old 5XFAD/Tg4-42^hom^ mice, Aβ concentrations were analyzed in the entire hippocampus with the newly established immunoassays to obtain quantitative information and to support the immunohistochemical analyses (Fig. 3). TBS- and SDS-soluble protein fractions were extracted from frozen hippocampal tissues and Aβ1-x and Aβ4-x peptides were compared between 5XFAD and 5XFAD/Tg4-42^hom^ mice. Aβ1-x concentrations in the hippocampus were comparable between 5XFAD and 5XFAD/Tg4-42^hom^ mice, both in the TBS-soluble (Fig. 3A) and SDS-soluble (Fig. 3C) fractions. Aβ4-x represented only a minor fraction of the Aβ species in the 5XFAD line. No changes in Aβ4-x levels were found in the TBS-soluble fraction (Fig. 3B). However, in the SDS fraction of 5XFAD/Tg4-42^hom^ mice, Aβ4-x appeared to be increased compared to 5XFAD littermates; however, without reaching statistical significance (*p* = 0.093; Fig. 3D). This increase cannot be ascribed to the transgene encoding the Aβ4–42 peptide expressed in the Tg4-42^hom^ line as Aβ4-x levels were below the detection limit in this line. It is further unlikely that the observed changes were the result of altered transgene expression after the crossing of 5XFAD and Tg4-42^hom^ mice, as mRNA expression levels of both the human APP and Aβ4–42 transgenes were unchanged in 5XFAD/Tg4-42^hom^ mice (Suppl. Fig. S4). To further investigate the Aβ-content in individual plaques, 6-month-old 5XFAD and 5XFAD/Tg4-42^hom^ mice were stained with 18H6 and D3E10 to detect Aβ4-x and Aβ42 peptides respectively. The relative Aβ4-x content of individual plaques in the cortex, hippocampus, and CA1 region was quantified and did not show significant differences. However, when the analysis was restricted to the border area of plaques in the CA1 region, where Aβ4–42 is mainly expressed in the Tg4-42^hom^ line, a significant increase in Aβ4-x content was detected in the 5XFAD/Tg4-42^hom^ line (Fig. 3E–-J).

### Amyloid Plaque Pathology Rescues Recognition but not Spatial Memory Deficits in 5XFAD/Tg4-42^hom^ Mice

Learning and memory of 5XFAD/Tg4-42^hom^ mice were tested at 3.5 and 6 months of age with the Novel Object Recognition (NOR) and the Morris Water Maze (MWM) tasks. Recognition memory was assessed with the NOR test. Twenty-four hours after a habituation trial (Open Field), the mice performed a training trial (Day 1) in which they were exposed to two identical objects.

**Fig. 4:**
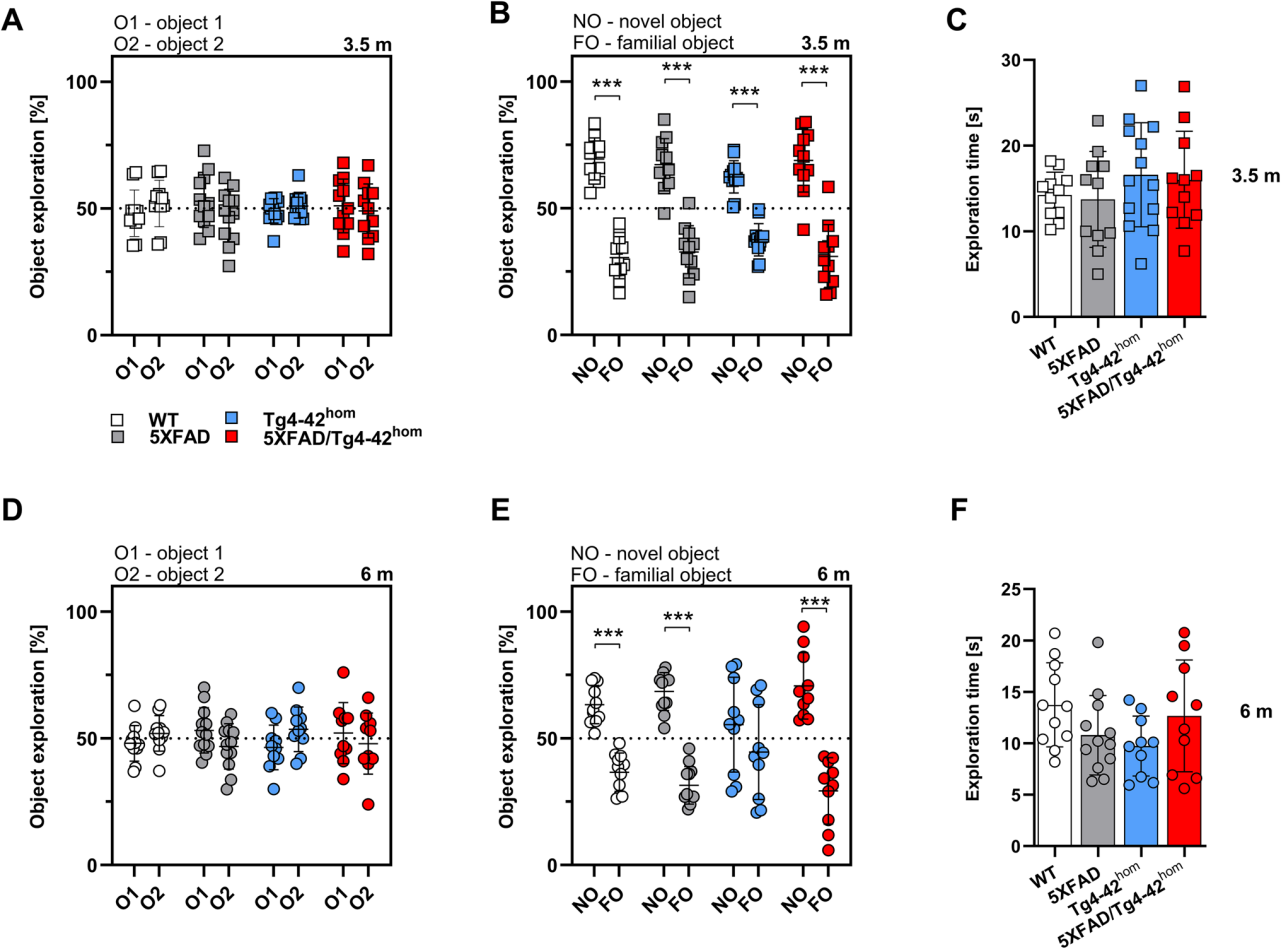
Object recognition memory. Female WT, 5XFAD, Tg4-42^hom^, and 5XFAD/Tg4-42^hom^ mice (*n* = 10–13) were tested at 3.5 (**A**–**C**) and 6 (**D**–**F**) months of age. No recognition memory deficits were observed at 3.5 months for WT and the three transgenic lines, as all showed similar exploration of the two identical objects (O1, O2) in the training trial (**A**) and significantly more exploration of the novel object (NO) compared to the familiar one (FO) in the probe trial (**B**). At 6 months, Tg4-42^hom^ mice displayed recognition memory deficits, with an equal exploration time of the novel and familiar object in the test trial on day 2, while 5XFAD/Tg4-42^hom^ mice showed a clear preference towards the novel object (**D**, **E**). No differences in exploration time were detected between the different lines at 3.5 or 6 months of age (**C**, **F**). All data are given as means ± SD. Dotted line represents 50% chance level. Two-way ANOVA followed by Bonferroni’s multiple comparison test (**A**, **B**, **D**, **E**). One-way ANOVA followed by Bonferroni’s multiple comparison test (**C**, **F**): ***p < 0.001. O1, object 1; O2, object 2; NO, novel object; FO, familiar object

All the transgenic lines and WT controls explored the two objects equally during the 5-min trial and showed no preference, neither at 3.5 (Fig. 4A) nor 6 (Fig. 4D) months of age. During the test trial (Day 2), performed 24 h after the training trial, one of the identical objects was replaced with a new object. At an age of 3.5 months, the three transgenic lines as well as the WT group showed intact recognition memory, displaying a significant preference for the novel object (Fig. 4B). As shown previously [41], the Tg4-42^hom^ group displayed recognition memory deficits at 6 months of age. During the test trial, Tg4-42^hom^ mice did not differentiate between the novel and the familiar object and explored them for an equal amount of time. Surprisingly, recognition memory was rescued in 6-month-old 5XFAD/Tg4-42^hom^ mice. This group preferentially explored the novel object, comparably to 5XFAD and WT, thus showing no recognition memory deficit at this time point (Fig. 4E). Total exploration times during the second day of the task were compared to exclude a possible influence of exploration time on the outcome of the task. No statistically significant differences between the genotypes were observed at either age (Fig. 4C, F).

The MWM test was performed to assess spatial memory. At first, 3 days of cue training allowed for the detection of possible sensory and motor deficits in this task. During this phase, the platform was tagged with a visible flag, and only distal visual cues were present. The average escape latency and the average speed in four trials with a maximum duration of 60 s were measured for each day. At 3.5 months, both WT and the three transgenic lines displayed an expected time-dependent reduction of latency to reach the platform, and no statistically significant differences were observed between the groups (Suppl. Fig. S5A). At this time point, statistically significant differences in the average speed were recorded among the lines (Suppl. Fig. S5B). WT and 5XFAD/Tg4-42^hom^ mice swam comparably and significantly slower than 5XFAD and Tg4-42^hom^ mice (*p* < 0.001). Wildtype, 5XFAD, and Tg4-42^hom^ mice showed a time-dependent reduction of the time necessary to reach the visible platform also at the 6 months’ time point (Suppl. Fig. S5C). This reduction was not observed in the 5XFAD/Tg4-42^hom^ line, which needed a significantly longer time to reach the platform during the 60 s trials compared to both WT mice (*p* < 0.001) and the two parental lines (*p* < 0.01). The altered behavior in 5XFAD/Tg4-42^hom^ was accompanied by a statistically significant reduced average speed compared to the other groups (WT and Tg4-42^hom^: *p* < 0.01; 5XFAD: *p* < 0.05) (Suppl. Fig. S5D).

During the five days of acquisition training, the platform was no longer visible and additional proximal visual cues were added. At 3.5 months of age, both 5XFAD and 5XFAD/Tg4-42^hom^ mice displayed an increased average escape latency compared to WT (*p* < 0.001), while no alterations were found in Tg4-42^hom^ animals, as previously reported [42] (Suppl. Fig. S5E). 5XFAD/Tg4-42^hom^ animals also took more time to reach the hidden platform compared to the Tg4-42^hom^ line (*p* < 0.05). During the acquisition training, young WT mice swam significantly slower than 5XFAD (*p* < 0.001) and Tg4-42^hom^ (*p* < 0.05) mice, respectively (Suppl. Fig. S5F). At six months of age, Tg4-42^hom^ mice displayed spatial learning deficits compared to WT littermates, showing an increased escape latency (*p* < 0.001) (Suppl. Fig. S5G). A comparable increase was observed in 5XFAD/Tg4-42^hom^ mice as well, which behaved differently from WT (*p* < 0.001) and 5XFAD (*p* < 0.001) littermates, which presented no alterations. No changes in average speed were detected in the acquisition phase at this time point (Suppl. Fig. S5H). Information on within-day group comparisons of the escape latencies at 3.5 and 6 months of age can be found in Suppl. Tables S2 and S3, respectively.

**Fig. 5:**
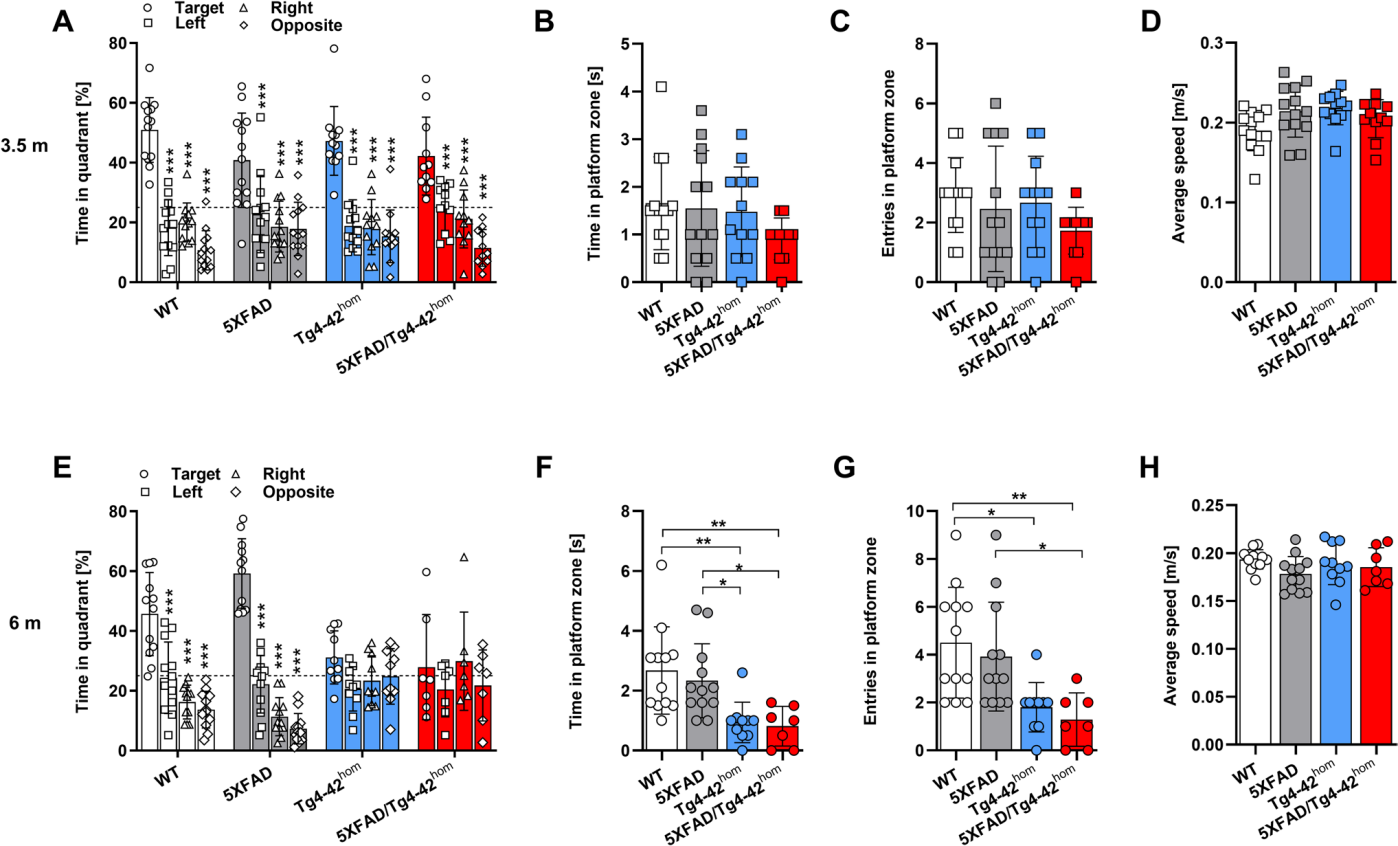
Morris Water Maze task: spatial reference memory in the probe trial in 3.5- and 6-month-old transgenic and WT control mice. Female WT, 5XFAD, Tg4-42^hom^, and 5XFAD/Tg4-42^hom^ mice (*n* = 7–13) were tested at 3.5 (**A**–**D**) and 6 (**E**–**H**) months of age. At 3.5 months, no spatial memory deficits were observed in any group (**A**), while at 6 months, a deficit was clearly present in Tg4-42^hom^ and 5XFAD/Tg4-42^hom^ mice (**E**). The number of entries and time in the platform zone were unchanged at 3.5 m (**B**, **C**), but significantly reduced in Tg4-42^hom^ and 5XFAD/Tg4-42hom mice at 6 m (**F**, **G**). No differences were observed in swimming speed at either time point (**D**, **H**). All data are given as means ± SD. Two-way ANOVA followed by Bonferroni’s multiple comparison test: *p < 0.05, **p < 0.01, ***p < 0.001

A single-trial probe test, during which the platform was removed, was performed 24 h after the end of the acquisition training to test for spatial memory impairments. The time spent in each quadrant, the time spent in the platform zone and the average speed were recorded. At 3.5 months, all analyzed groups showed no obvious spatial memory deficits as they spent statistically significantly more time swimming in the target compared to the other quadrants (Fig. 5A). At 6 months, both WT and 5XFAD animals showed no spatial memory deficits (Fig. 5E). As expected [42, 43], Tg4-42^hom^ mice explored the four quadrants for an equal amount of time, indicating the presence of a memory deficit at this time point. A spatial memory impairment could also be observed in 5XFAD/Tg4-42^hom^ mice, as they failed to remember the location of the target quadrant. The performances of the four different lines were directly compared by recording the times and entries in the target quadrant and the platform zone. In young animals, no significant differences were observed in either the time or entries in the platform zone (Fig. 5B, C). At 6 months, anticipated by the inability to remember the position of the target quadrant, Tg4-42^hom^ and 5XFAD/Tg4-42^hom^ mice showed decreased numbers of entries into the platform zone. Both Tg4-42^hom^ and 5XFAD/Tg4-42^hom^ mice spent less time in the platform zone compared to WT (*p* < 0.01) and 5XFAD (*p* < 0.05) animals (Fig. 5F) and showed fewer entries into the platform area (Fig. 5G). At both time points, no alterations in the average speed were detected in the probe trial (Fig. 5D, H).

### Extracellular Amyloid Pathology Ameliorates CA1 Neuron Loss in 5XFAD/Tg4-42^hom^ Mice

**Fig. 6:**
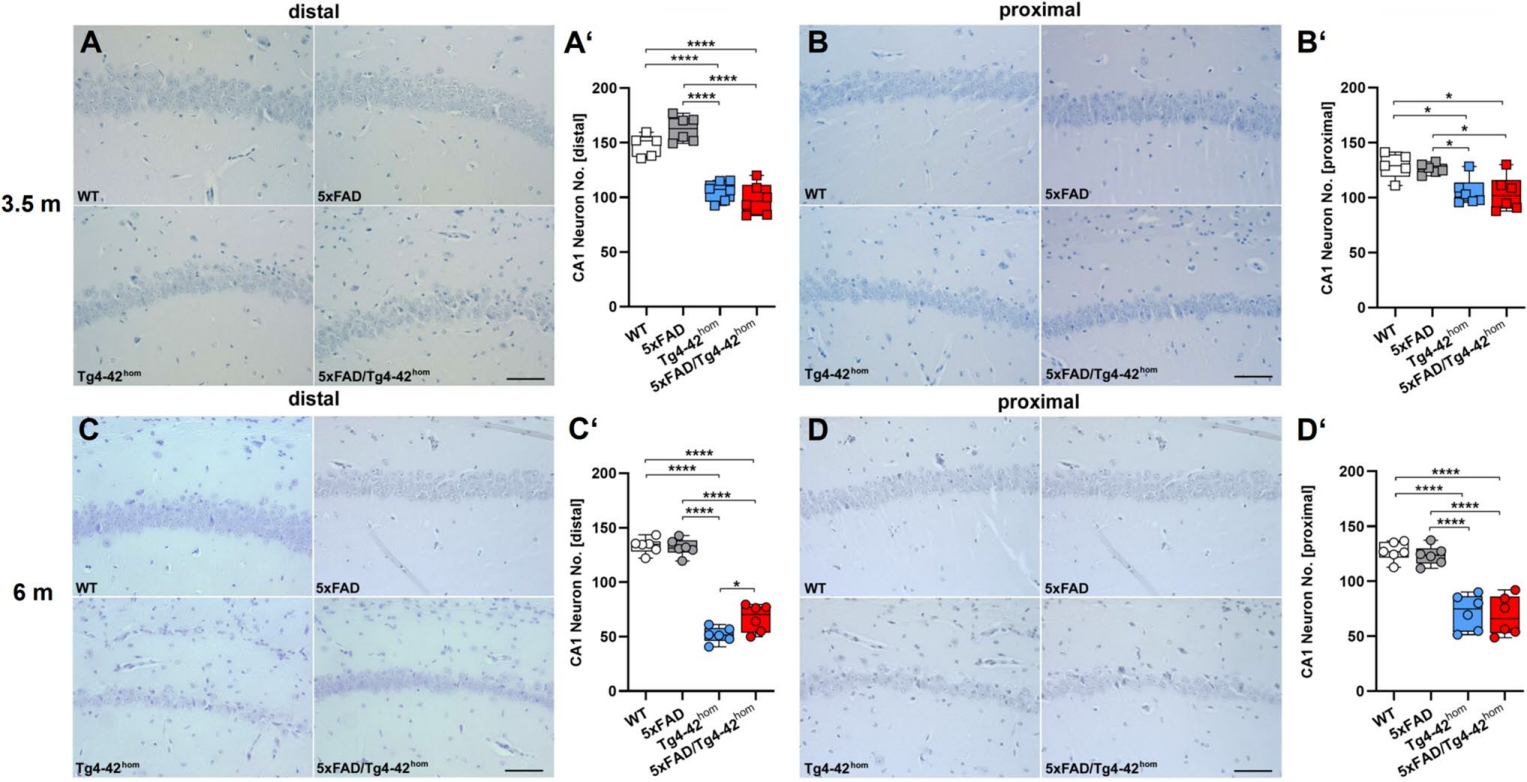
Distal and proximal CA1 neuron loss in 3.5- and 6-month-old mice. Sagittal paraffin brain sections from female WT, 5XFAD, Tg4-42^hom^, and 5XFAD/Tg4-42^hom^ mice (*n* = 5–6) were stained with hematoxylin. Young Tg4-42^hom^ and 5XFAD/Tg4-42^hom^ mice showed significantly reduced distal and proximal CA1 neuron numbers (**A**–**B**′). At 6 months, 5XFAD/Tg4-42^hom^ animals showed higher distal CA1 but not proximal CA1 neuron numbers compared to Tg4-42^hom^ littermates (**C**–**D**′). All data are given as means ± SD. One-way ANOVA followed by Bonferroni’s multiple comparison test: *p < 0.05, ***p < 0.001

Neuron loss in the CA1 region of the hippocampus is observed in a time- and genotype-dependent manner in the Tg4-42^hom^ line and is associated with behavioral deficits observed in the NOR and MWM behavioral tasks [27, 41–43]. Hematoxylin-stained distal and proximal CA1 pyramidal neurons were counted to assess whether the presence of amyloid plaques in 5XFAD/Tg4-42^hom^ mice might impact the neuron loss observed in the Tg4-42^hom^ mouse model (Fig. 6). Neuron loss was detected already in young animals (Fig. 6A, B), as both Tg4-42^hom^ and 5XFAD/Tg4-42^hom^ mice displayed ~ 30% reduction in the distal and ~ 20% reduction in the proximal part of CA1 compared to WT littermates (a loss being statistically significant compared to both WT and 5XFAD animals; distal CA1: *p* < 0.001, proximal CA1: *p* < 0.05) (Fig. 6A′, B′). As expected, an age-dependent aggravation of the neuron loss was observed in the Tg4-42^hom^ line, reaching ~ 60% in the distal CA1 and ~ 45% in the proximal CA1 compared to the WT group in 6-month-old animals (Fig. 6C′, D′). Confirming recent findings, the neuron loss was more prominent towards the distal region [41]. While 5XFAD/Tg4-42^hom^ mice showed a comparable loss in proximal pyramidal CA1 neurons (Fig. 6D), a reduced loss was observed in the distal part, as the filial line presented with significantly higher neuron numbers compared to Tg4-42^hom^ littermates (*p* < 0.05) (Fig. 6C). At both time points, no loss of CA1 pyramidal neurons was observed in the 5XFAD line, confirming previous findings [44].

Tg4-42^hom^ and 5XFAD/Tg4-42^hom^ mice presented with a comparable and statistically significant reduction of the CA1 and pyramidal layer areas when contrasted to WT and 5XFAD littermates at 3.5 months of age (Suppl. Fig. S6A, B). At 6 months, the Tg4-42^hom^ mice consistently displayed a smaller pyramidal layer and CA1 area compared to WT and 5XFAD mice (Suppl. Fig. S6C, D). At this time point, 5XFAD/Tg4-42^hom^ mice showed no difference in the CA1 area compared to WT (Suppl. Fig. S6C). However, conforming to the higher distal CA1 neuron numbers, the pyramidal layer area in aged 5XFAD/Tg4-42^hom^ mice was significantly larger compared to Tg4-42^hom^ littermates (*p* < 0.05), despite both lines having a significantly smaller area compared to WT and 5XFAD littermates (*p* < 0.001) (Suppl. Fig. S6D).

### No Overt Alterations in the Neuroinflammatory Phenotype Between 5XFAD and 5XFAD/Tg4-42^hom^ Mice

**Fig. 7:**
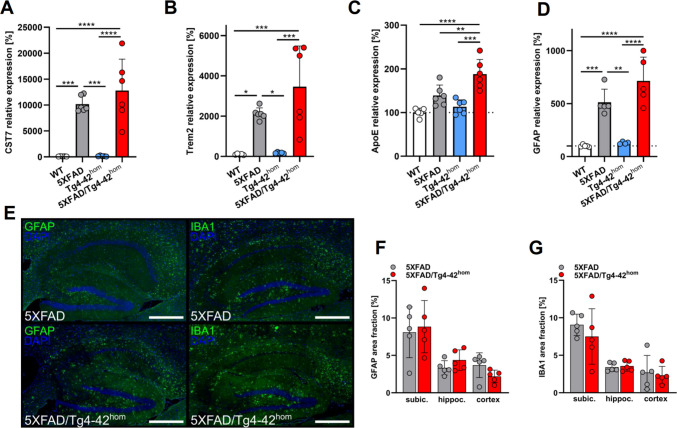
Quantitative Real-Time PCR analysis of the neuroinflammatory markers *CST7* (**A**), *TREM2* (**B**), *APOE* (**C**), and *GFAP* (**D**) in the hippocampus of WT, 5XFAD, Tg4-42^hom^, and 5XFAD/Tg4-42^hom^ mice at 6 months of age (*n *= 5–6). Sagittal brain sections from 6-month-old female 5XFAD and 5XFAD/Tg4-42^hom^ mice were stained with GFAP and IBA1 antibodies (**E**–**G**), revealing unchanged levels between 5 and 5XFAD/Tg4-42^hom^ mice (*n* = 5). All data are given as means ± SD. Unpaired *t*-test

Finally, we assessed whether the co-expression of Aβ4–42 altered the neuroinflammatory phenotype of 5XFAD mice in 6-month-old animals. Quantitative Real-Time PCR analyses of hippocampal samples from all four genotypes were carried out, and micro- and astroglia markers *CST7* (Fig. 7A), *TREM2* (Fig. 7B), *APOE* (Fig. 7C) and *GFAP* (Fig. 7D) were measured. As expected, plaque-bearing 5XFAD and 5XFAD/Tg4-42^hom^ mice showed statistically significantly increased *CST7*, *TREM2* and *GFAP* expression levels compared to WT or Tg4-42^hom^, while levels in 5XFAD/Tg4-42^hom^ mice were not different from 5XFAD mice. In the case of *APOE*, 5XFAD/Tg4-42^hom^ mice showed significantly increased expression levels compared to all other groups. In addition, sagittal paraffin brain sections were stained with antibodies against the astrocytic and microglial markers GFAP and IBA1 and quantifications were performed in the subiculum, hippocampus and cortex. No significant differences with regard to micro- and astrogliosis were observed between 5XFAD and 5XFAD/Tg4-42^hom^ mice in any of the brain regions analyzed (Fig. 7E–G).

## Discussion

In rodent models of AD, hippocampal neuron loss has been associated with the development of memory deficits. Damage limited to the hippocampus was sufficient to cause recognition memory deficits, and this has been tested in the NOR task in rodents, non-human primates, and memory-impaired patients [45, 46]. In Tg4-42^hom^ mice, a ~ 50% neuron loss at 6 months of age was accompanied by clear spatial memory deficits in the MWM task, while 5-month-old mice, presenting with ~ 43% of CA1 pyramidal neuron loss, showed only mild spatial learning deficits and intact spatial recognition memory [42]. In agreement, preservation of 10–20% of neurons resulted in restored spatial and recognition memory in Tg4-42^hom^ mice at 6 months [41]. Due to the used genetic construct, these mice only produce Aβ4–42 peptides in the absence of APP overexpression, since Aβ is linked to the thyrotropin-releasing hormone. Therefore, Aβ4–42 is generated by prohormone convertases along the secretory pathway independently of β- and γ-secretase processing [27]. In the present study, we observed the previously reported recognition and spatial memory deficits in 6-month-old Tg4-42^hom^ mice when tested in the MWM and NOR tasks, accompanied by the age-dependent loss of CA1 pyramidal neurons [41, 47]. As the probe trial performance in Tg4-42^hom^ and 5XFAD/Tg4-42^hom^ is poor at 6 months of age, this task would primarily detect a rescuing effect in these mice at this age. However, a potential aggravation would have been obvious at the earlier time point, when neither 5XFAD nor Tg4-42^hom^ show an impairment. No altered phenotype was found in 6-month-old 5XFAD mice in the MWM or novel object recognition tasks which is in line with data published by others [48, 49]. Different regions of the CA1 pyramidal layer have been correlated with the development of specific memory deficits: the distal CA1 is primarily linked to non-spatial memory, while the proximal part is associated with spatial memory formation [50]. We chose 3.5 and 6 months of age as time points for investigation, as, e.g., spatial memory deficits have been described in 5XFAD mice beyond 6 months of age [49] and therefore, any potential protective effects of the presence of plaques would have been difficult to detect with behavioral read-outs. Upon crossing of the 5XFAD and Tg4-42^hom^ lines, no further aggravation of spatial learning and memory deficits was detected in the 5XFAD/Tg4-42^hom^ line as they performed comparably to Tg4-42^hom^ littermates at both time points. Intriguingly, recognition memory deficits in Tg4-42^hom^ mice appeared to be rescued after crossing to 5XFAD mice. The observations with regard to neuron loss in the distal and proximal pyramidal layers of 5XFAD/Tg4-42^hom^ mice at 6 months are fully consistent with the outcomes of the behavioral tasks. The hippocampus shows some heterogeneity of pyramidal neurons with regard to memory-guided behaviors, which is seen across the transverse (proximo-distal), radial (deep-superficial) and longitudinal (dorsal-ventral) principal anatomical axes [50], with neurons in the distal CA1 (towards subiculum) displaying higher tuning for objects and odors [50, 51]. 5XFAD/Tg4-42^hom^ mice displayed decreased neuron loss compared to Tg4-42^hom^ mice in the distal portion of the pyramidal layer. As this region is more associated with non-spatial memory [50], it may be linked to the lack of recognition memory deficits of the bigenic line in the NOR task. Conversely, the comparable loss of neurons in the proximal CA1 region, which is mainly associated with spatial memory, might correlate with the deficits found in the MWM test for both lines. 5XFAD mice, on the other hand, presented with intact spatial and recognition memory at 6 months and no loss of CA1 pyramidal neurons. Previous studies also reported no loss of hippocampal neurons in this line, even at 12 months of age [44]. As a result of neurodegeneration in AD patients, robust brain atrophy is observed and is considered one of the most prominent neuropathological hallmarks of AD [52]. In rodent models, a reduced hippocampal volume has been associated with spatial memory deficits in several studies [53, 54]. In further support of a link between the partial preservation of neuron numbers in the proximal CA1 and the rescue of recognition memory deficits, the pyramidal layer area in 6-month-old 5XFAD/Tg4-42^hom^ mice showed less atrophy compared to Tg4-42^hom^ littermates.

In the Tg4-42^hom^ line, neuron loss is primarily attributed to the intracellular accumulation of Aβ4–42 oligomers in CA1 pyramidal neurons [27]. In contrast, the 5XFAD line shows extracellular amyloid plaques in the hippocampus but neither intracellular Aβ accumulation nor neuron loss in the CA1 region [44]. No changes in the extracellular amyloid pathology were observed in the CA1 region by immunohistochemical analysis in young or aged 5XFAD/Tg4-42^hom^ mice compared to the 5XFAD line, as the amyloid plaque load for both full-length and N-terminal-truncated Aβ4-x peptides remained unaffected. In good agreement, neuroinflammatory markers were as well largely unchanged between 5XFAD and 5XFAD/Tg4-42^hom^ mice in the hippocampus. In addition, our data clearly show that the additional extracellular amyloid pathology introduced through the 5XFAD background did not aggravate the spatial impairments or the neuron loss of Tg4-42^hom^ mice. Given the reduced neuron loss and the improvement in recognition memory deficits in 5XFAD/Tg4-42^hom^ mice at 6 months of age, these findings align with the idea that insoluble amyloid plaques could have some buffering capacity [14, 18], possibly capturing soluble Aβ4–42 peptides and reducing their associated neurotoxicity. While the subtle differences we observed in the pathology of the double transgenic mice are insufficient to conclusively support this hypothesis, our data suggest that the presence of insoluble plaques does not exacerbate cognition nor increase loss of neurons in the CA1 region of the hippocampus.

The observation of increased Aβ4-x immunoreactivity in the border area of plaques in the CA1 region (where Aβ4–42 is mainly expressed in the Tg4-42^hom^ transgenic line) would support such a mechanism. Soluble Aβ conformations are regarded as major neurotoxic species [8, 55] contributing to dementia in humans [14]. In line with this concept, compounds promoting the aggregation of Aβ peptides have been reported to reduce the amount of Aβ oligomers and to improve cognitive dysfunction in a transgenic mouse model of AD [56].

In conclusion, our results support the importance of soluble Aβ variants in the pathogenesis of AD, and suggest that sequestration of oligomeric Aβ species into amyloid plaques might, at least in the early phases of the disease, reduce their lethal toxicity.

## Supplementary Information

Below is the link to the electronic supplementary material.

ESM 1 (DOCX 1.56 MB)

## Data Availability

Datasets generated within the current study are included or are available from the corresponding author on reasonable request.
