# Supplementary material for: Amyloid Plaques Ameliorate Memory Deficits and Hippocampal Neuron Loss in an Aβ4-42-Driven Alzheimer’s Disease Mouse Model
Source: Mol Neurobiol. 2026 May 15;63(1):633. doi: 10.1007/s12035-026-05912-x (PMC13179277; doi:10.1007/s12035-026-05912-x)
Supplement: Supplementary file 1 — (DOCX 1.56 MB) [file 12035_2026_5912_MOESM1_ESM.docx]

**Supplementary Information**

**Zampar et al.: Extracellular amyloid plaques ameliorate memory deficits and hippocampal neuron loss in an Alzheimer’s disease mouse model driven by soluble Aβ4-42 peptides**

**
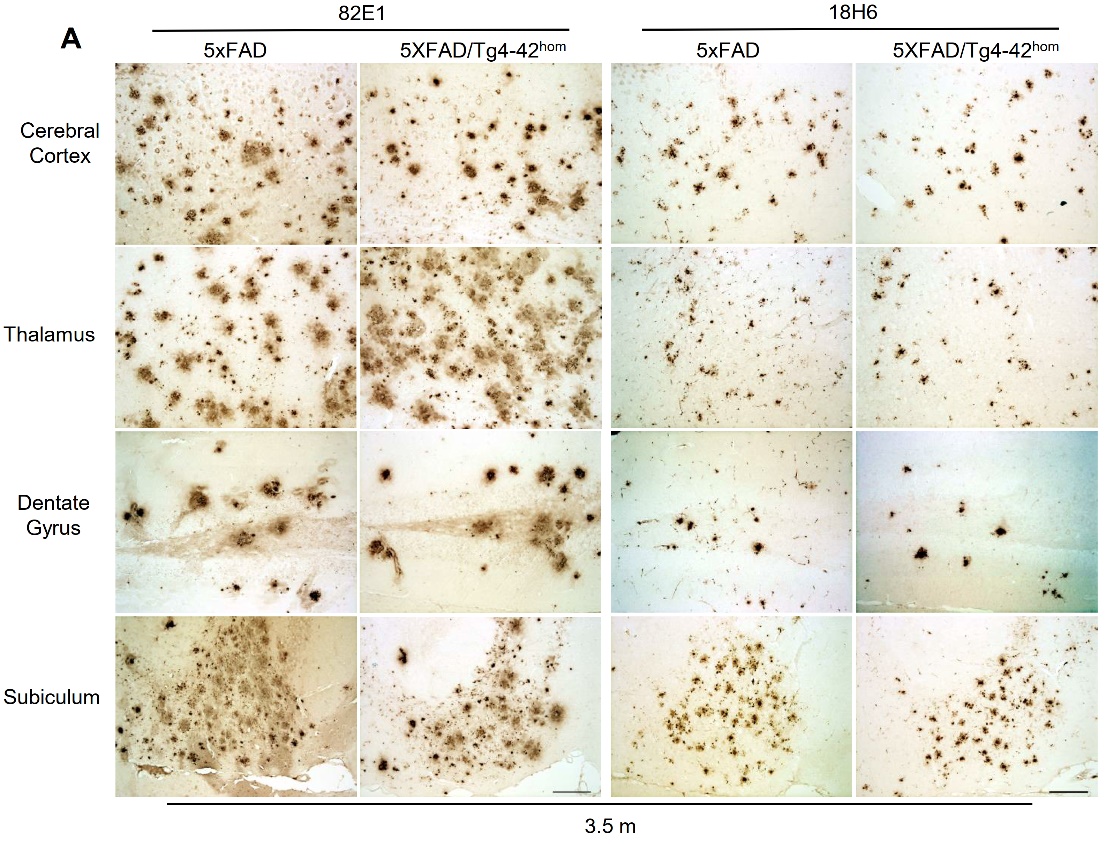
**

**
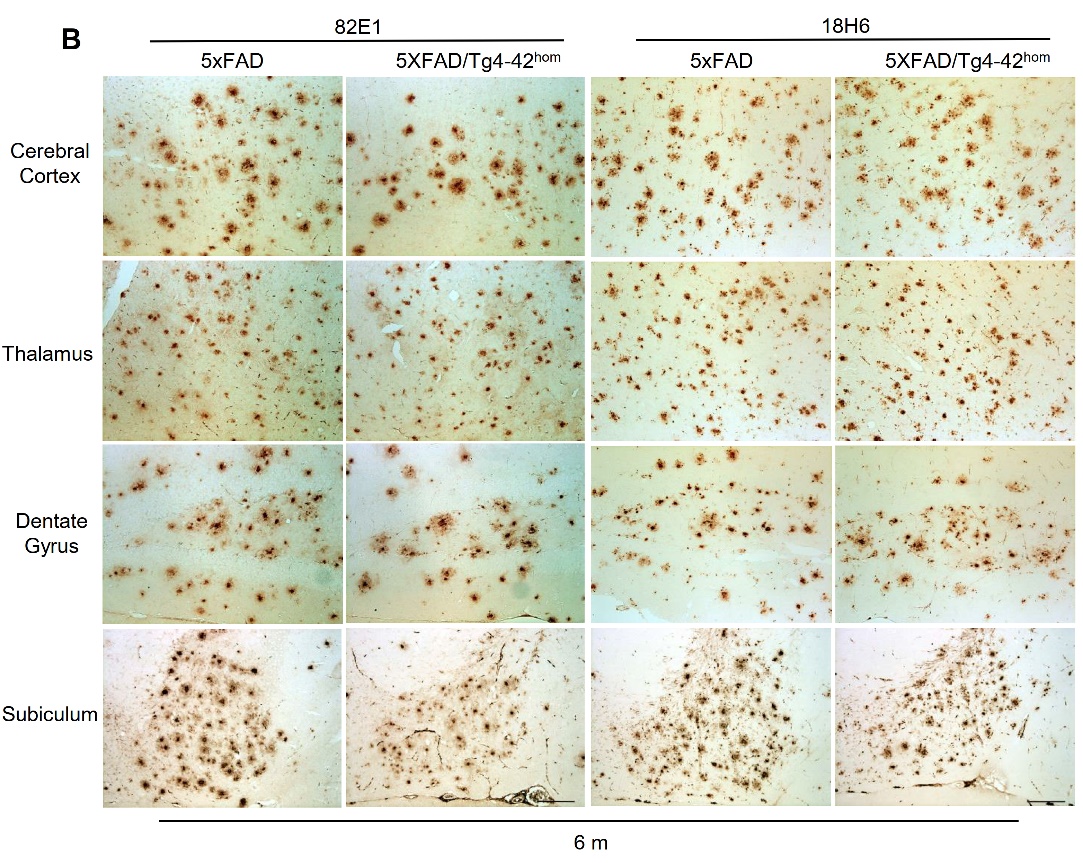
**

**Suppl. Fig. S1:** Representative images depicting amyloid plaque pathology assessed with 82E1 (Aβ1-x) and 18H6 (Aβ4-x) antibodies in 3.5- (**A**) and 6-month-old 5xFAD and 5xFAD/Tg4-42hom mice (**B**). Scale bar: 100 µm

**Development Aβ1-x and Aβ4-x electrochemiluminescence immunoassays**

#### Aliquots of mAb 18H6 (specific towards Aβ4-x peptides) and mAb 82E1 (specific towards Aβ1-x peptides, RRID:AB_10707424, IBL International) were conjugated with biotin to serve as capture antibodies. After blocking unspecific binding sites, 96-well small spot streptavidin plates were coated with biotinylated mAb 18H6 or mAb 82E1 antibodies and paired with Sulfo-TAG mAb 4G8 (#D20RQ-2, MSD) as detection antibody and a series of validation experiments were carried out. Synthetic Aβ1-40 and Aβ4-40 peptides were used in the Aβ1-x and Aβ4-x specific assays, respectively. To optimize the detection of the Aβ peptides, different concentrations of biotinylated mAb 18H6 and mAb 82E1 capture antibody during plate coating were tested (capture antibody titration). While we observed an improvement in the detection sensitivity by increasing the concentration of the capture antibody for mAb 18H6-bio (Suppl. Fig. S2B) from 0.4-0.5 µg/mL to 1 µg/mL and 2 µg/mL, increasing the concentration of mAb 82E1-bio (Suppl. Fig. S2A) above 1 µg/mL reduced the detection sensitivity. In light of the measured detection sensitivity and to balance the costs of the antibodies, the final working concentrations for the detection and capture antibodies was 1 μg/mL for mAb 82E1-bio, while 18H6-bio was used at a concentration of 0.6 μg/mL.

**
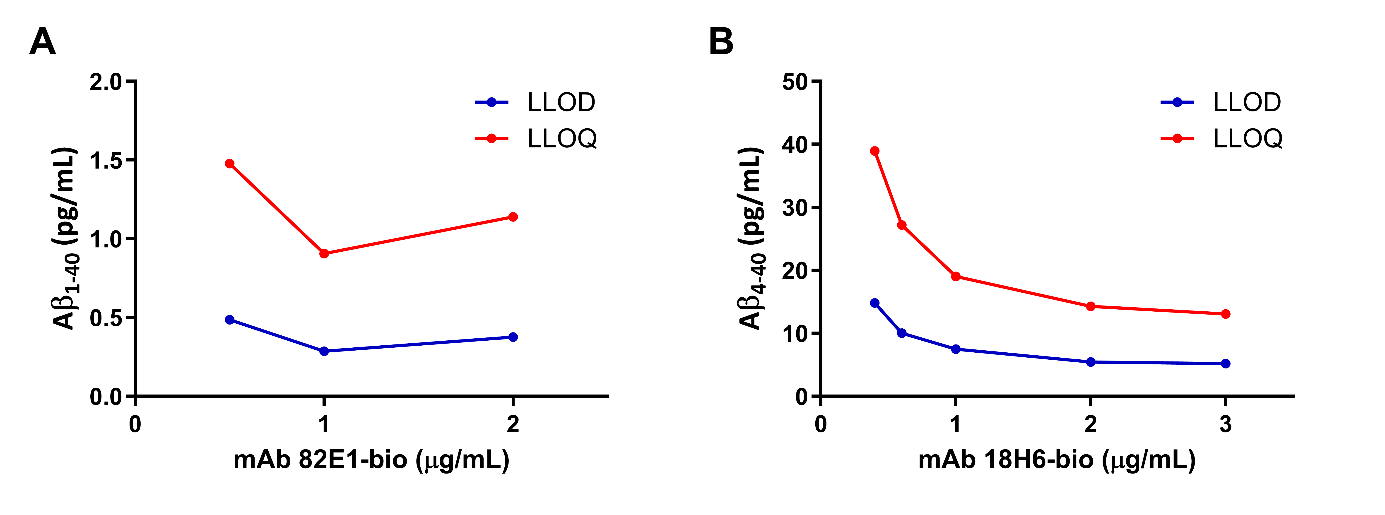
**

**Suppl. Fig. S2:** Capture antibody titration for biotinylated 82E1 (A) and 18H6 (B).

**Lower Limit of Detection, Lower Limit of Quantification, and Upper Limit of Quantification**

The measurements in each assay run were extrapolated from the fitting of a four-parameter logistic standard curve with the MSD Discovery Workbench 4.0.12 software. The raw signals were obtained from seven fourfold serial dilutions of the synthetic Aβ calibrator peptides plus a zero calibrator (blank). An initial extended fourfold calibrator dilution series including ten fourfold serial dilutions starting at 120 ng/ml was analyzed to determine the upper limit of quantification (ULOQ) and the appropriate standard curve range for each assay (**Suppl. Fig. S3**). The ULOQ was measured in a single assay and is defined as the highest calibrator concentration that showed < 20% coefficient of variation (CV) between two technical replicates (regarding both signal and calculated concentration) and 85–115% recovery (i.e., the calculated concentration had to be in the range of 85–115% of the theoretical calibrator concentration). Additionally, the LLOD was calculated for each single assay plate as the lowest analyte concentration generating a signal three SDs above the lowest standard/blank with the option “use minimum error estimates” activated. The lower limit of quantification (LLOQ) was defined as the lowest analyte concentration producing a signal 10 SDs above the lowest standard/blank. ULOQs and mean LLODs and LLOQs, calculated from n = 4-5 assay runs, are summarized in **Table S1**. The starting concentration of the fourfold dilution Aβ1-40 standard curve in in the Aβ1-x specific was chosen at 7500 pg/ml, while 30000 pg/ml was the starting concentration of the Aβ4-40 calibrator used in the Aβ4-x specific assays.


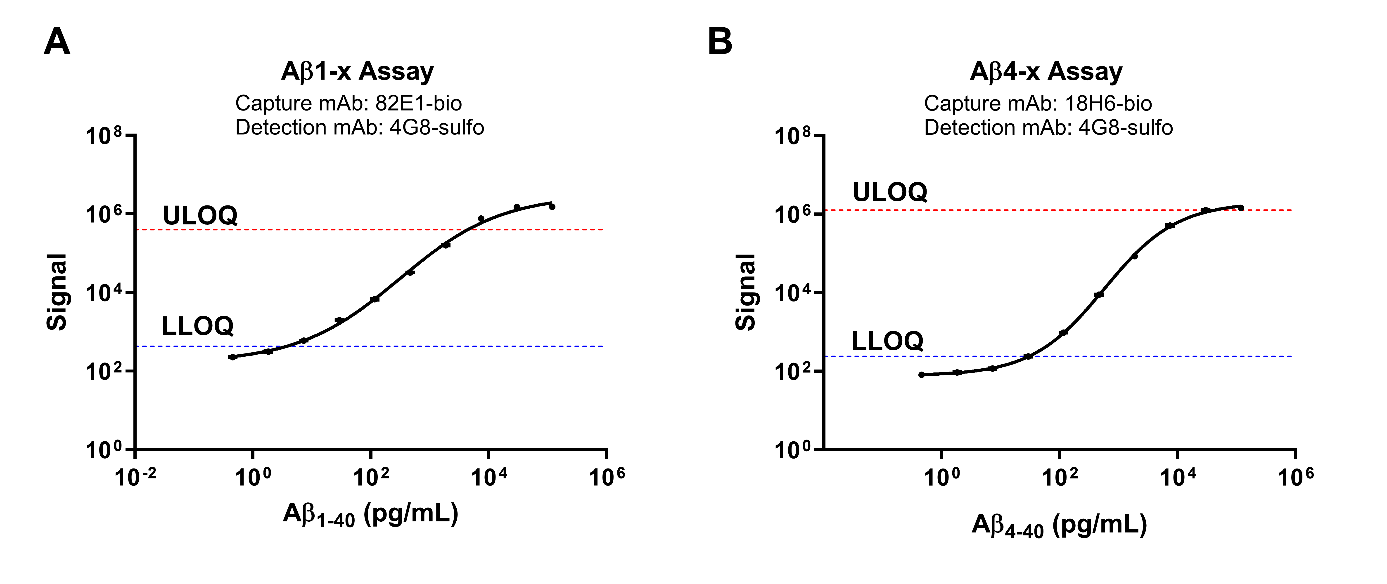


**Suppl. Fig. S3**: Extended standard curves of the Aβ-specific assays, showing the lower and upper limits of quantification

| **Aβ assay** | **Capture mAb-Biotin** | **Detection mAb-sulfo-Tag** | **LLOD** | | **LLOQ** | | **ULOD** |
| --- | --- | --- | --- | --- | --- | --- | --- |
|  |  |  | pg/mL | SD | pg/mL | SD | pg/mL |
| Aβ1-x | 82E1 (1 μg/ml) | 4G8 (1:50) | 0.87 | 0.37 | 2.52 | 1.03 | 5600 |
| Aβ4-x | 18H6 (0.6 μg/ml) | 4G8 (1:50) | 12.64 | 1.81 | 33.42 | 1.20 | 30000 |

**Suppl. Table S1:** Average LLODs, LLOQs and ULOQ of the Aβ-specific immunoassays


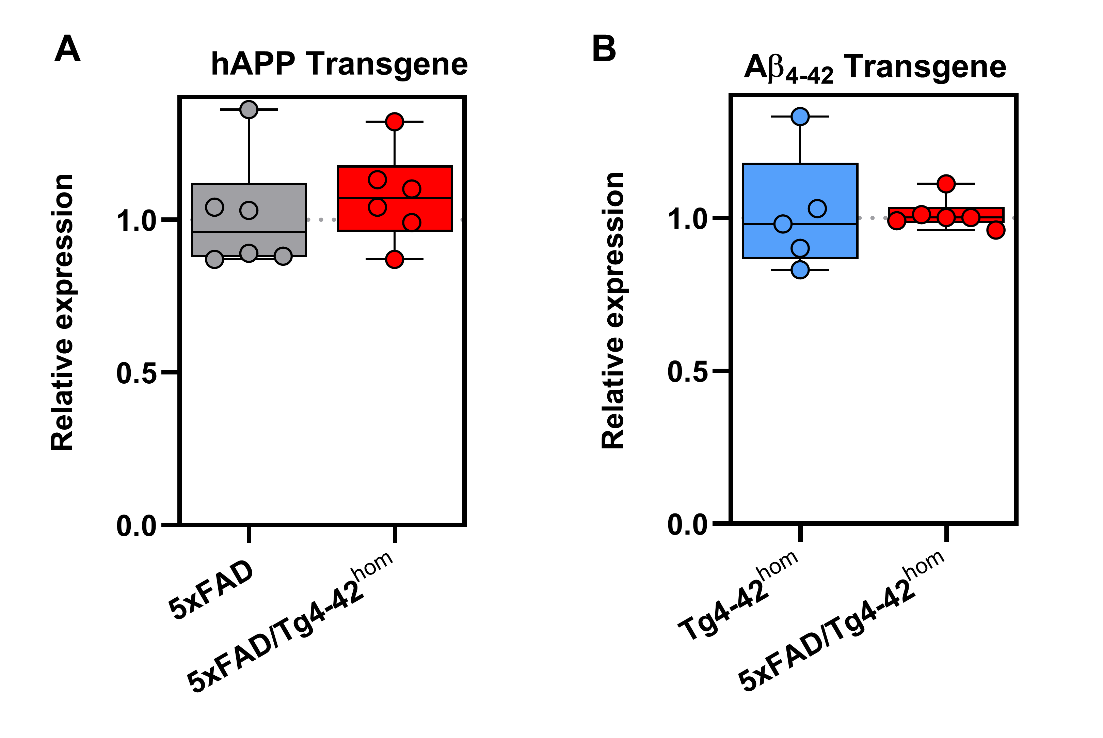


**Suppl. Fig. S4:** Transgene expression of hAPP (A) and the transgenic Aβ4-42 construct (B) determined by qRT-PCR in the hippocampus at 6 months of age.

**
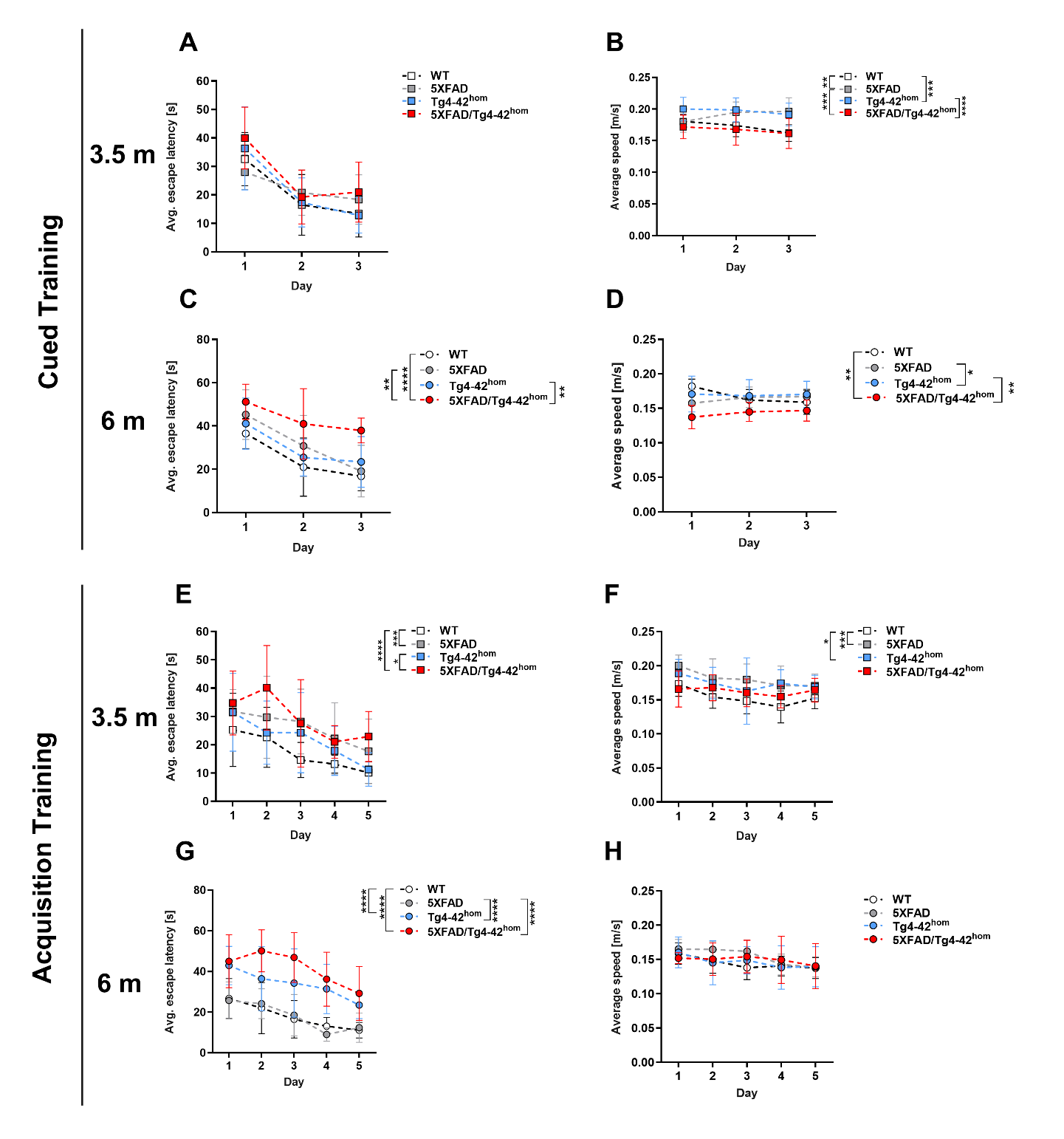
**

**Suppl. Fig. S5:** Cued and acquisition training in the Morris Water Maze task at 3.5 and 6 months of age. All data are given as means ± SD. Two-way repeated measures ANOVA followed by Bonferroni’s multiple comparison test. **p* < 0.05; ***p* < 0.01, ****p* < 0.001, *****p* < 0.0001.

| **Within-day group comparison MWM 3.5 m:** | Predicted (LS) Mean diff. | 95.00% CI of diff. | Summary | Adjusted *p* value |
| --- | --- | --- | --- | --- |
|  |  |  |  |  |
| **Day 1** |  |  |  |  |
| WT vs. 5XFAD | -6.462 | -17.76 to 4.834 | ns | 0.7756 |
| WT vs. Tg4-42^hom^ | -6.195 | -17.72 to 5.334 | ns | 0.924 |
| WT vs. 5XFAD/Tg4-42^hom^ | -9.469 | -21.27 to 2.329 | ns | 0.2024 |
| 5XFAD vs. Tg4-42^hom^ | 0.2663 | -11.26 to 11.80 | ns | >0.9999 |
| 5XFAD vs. 5XFAD/Tg4-42^hom^ | -3.008 | -14.81 to 8.791 | ns | >0.9999 |
| Tg4-42^hom^ vs. 5XFAD/Tg4-42^hom^ | -3.274 | -15.30 to 8.747 | ns | >0.9999 |
|  |  |  |  |  |
| **Day 2** |  |  |  |  |
| WT vs. 5XFAD | -7.1 | -18.40 to 4.196 | ns | 0.5742 |
| WT vs. Tg4-42^hom^ | -1.724 | -13.25 to 9.805 | ns | >0.9999 |
| WT vs. 5XFAD/Tg4-42^hom^ | -17.5 | -29.30 to -5.703 | *** | 0.0006 |
| 5XFAD vs. Tg4-42^hom^ | 5.376 | -6.153 to 16.90 | ns | >0.9999 |
| 5XFAD vs. 5XFAD/Tg4-42^hom^ | -10.4 | -22.20 to 1.397 | ns | 0.1189 |
| Tg4-42^hom^ vs. 5XFAD/Tg4-42^hom^ | -15.78 | -27.80 to -3.755 | ** | 0.0034 |
|  |  |  |  |  |
| **Day 3** |  |  |  |  |
| WT vs. 5XFAD | -13.58 | -24.87 to -2.281 | ** | 0.0095 |
| WT vs. Tg4-42^hom^ | -9.652 | -21.18 to 1.877 | ns | 0.1611 |
| WT vs. 5XFAD/Tg4-42^hom^ | -12.96 | -24.76 to -1.162 | * | 0.0229 |
| 5XFAD vs. Tg4-42^hom^ | 3.925 | -7.604 to 15.45 | ns | >0.9999 |
| 5XFAD vs. 5XFAD/Tg4-42^hom^ | 0.6168 | -11.18 to 12.41 | ns | >0.9999 |
| Tg4-42^hom^ vs. 5XFAD/Tg4-42^hom^ | -3.308 | -15.33 to 8.713 | ns | >0.9999 |
|  |  |  |  |  |
| **Day 4** |  |  |  |  |
| WT vs. 5XFAD | -9.046 | -20.34 to 2.250 | ns | 0.2047 |
| WT vs. Tg4-42^hom^ | -4.718 | -16.25 to 6.811 | ns | >0.9999 |
| WT vs. 5XFAD/Tg4-42^hom^ | -7.871 | -19.67 to 3.927 | ns | 0.4627 |
| 5XFAD vs. Tg4-42^hom^ | 4.328 | -7.200 to 15.86 | ns | >0.9999 |
| 5XFAD vs. 5XFAD/Tg4-42^hom^ | 1.175 | -10.62 to 12.97 | ns | >0.9999 |
| Tg4-42^hom^ vs. 5XFAD/Tg4-42^hom^ | -3.154 | -15.17 to 8.868 | ns | >0.9999 |
|  |  |  |  |  |
| **Day 5** |  |  |  |  |
| WT vs. 5XFAD | -7.569 | -18.87 to 3.727 | ns | 0.455 |
| WT vs. Tg4-42^hom^ | -1.148 | -12.68 to 10.38 | ns | >0.9999 |
| WT vs. 5XFAD/Tg4-42^hom^ | -12.8 | -24.60 to -1.003 | * | 0.0255 |
| 5XFAD vs. Tg4-42^hom^ | 6.421 | -5.108 to 17.95 | ns | 0.8376 |
| 5XFAD vs. 5XFAD/Tg4-42^hom^ | -5.232 | -17.03 to 6.566 | ns | >0.9999 |
| Tg4-42^hom^ vs. 5XFAD/Tg4-42^hom^ | -11.65 | -23.67 to 0.3683 | ns | 0.0631 |

**Suppl. Table S2:** Within-day group comparison of latency in the MWM Acquisition trial at 3.5 months of age (2-way ANOVA with Bonferroni multiple comparisons test).

| **Within-day group comparisons MWM 6m:** | Predicted (LS) Mean diff. | 95.00% CI of diff. | Summary | Adjusted *p* value |
| --- | --- | --- | --- | --- |
|  |  |  |  |  |
| **Day 1** |  |  |  |  |
| WT vs. 5XFAD | 0.9417 | -10.22 to 12.10 | ns | >0.9999 |
| WT vs. Tg4-42^hom^ | -16.25 | -27.95 to -4.544 | ** | 0.0017 |
| WT vs. 5XFAD/Tg4-42^hom^ | -18.35 | -31.35 to -5.351 | ** | 0.0013 |
| 5XFAD vs. Tg4-42^hom^ | -17.19 | -28.89 to -5.486 | *** | 0.0008 |
| 5XFAD vs. 5XFAD/Tg4-42^hom^ | -19.29 | -32.29 to -6.292 | *** | 0.0006 |
| Tg4-42^hom^ vs. 5XFAD/Tg4-42^hom^ | -2.103 | -15.57 to 11.37 | ns | >0.9999 |
|  |  |  |  |  |
| **Day 2** |  |  |  |  |
| WT vs. 5XFAD | -2.144 | -13.30 to 9.015 | ns | >0.9999 |
| WT vs. Tg4-42^hom^ | -14.44 | -26.14 to -2.736 | ** | 0.0072 |
| WT vs. 5XFAD/Tg4-42^hom^ | -28.17 | -41.17 to -15.17 | **** | <0.0001 |
| 5XFAD vs. Tg4-42^hom^ | -12.3 | -24.00 to -0.5917 | * | 0.0337 |
| 5XFAD vs. 5XFAD/Tg4-42^hom^ | -26.03 | -39.03 to -13.03 | **** | <0.0001 |
| Tg4-42^hom^ vs. 5XFAD/Tg4-42^hom^ | -13.73 | -27.20 to -0.2606 | * | 0.0431 |
|  |  |  |  |  |
| **Day 3** |  |  |  |  |
| WT vs. 5XFAD | -1.996 | -13.16 to 9.164 | ns | >0.9999 |
| WT vs. Tg4-42^hom^ | -17.83 | -29.54 to -6.129 | *** | 0.0004 |
| WT vs. 5XFAD/Tg4-42^hom^ | -30.39 | -43.39 to -17.39 | **** | <0.0001 |
| 5XFAD vs. Tg4-42^hom^ | -15.84 | -27.54 to -4.133 | ** | 0.0024 |
| 5XFAD vs. 5XFAD/Tg4-42^hom^ | -28.4 | -41.40 to -15.40 | **** | <0.0001 |
| Tg4-42^hom^ vs. 5XFAD/Tg4-42^hom^ | -12.56 | -26.03 to 0.9101 | ns | 0.0827 |
|  |  |  |  |  |
| **Day 4** |  |  |  |  |
| WT vs. 5XFAD | 4.065 | -7.095 to 15.22 | ns | >0.9999 |
| WT vs. Tg4-42^hom^ | -18.33 | -30.03 to -6.621 | *** | 0.0003 |
| WT vs. 5XFAD/Tg4-42^hom^ | -23.15 | -36.15 to -10.15 | **** | <0.0001 |
| 5XFAD vs. Tg4-42^hom^ | -22.39 | -34.09 to -10.69 | **** | <0.0001 |
| 5XFAD vs. 5XFAD/Tg4-42^hom^ | -27.22 | -40.22 to -14.22 | **** | <0.0001 |
| Tg4-42^hom^ vs. 5XFAD/Tg4-42^hom^ | -4.829 | -18.30 to 8.642 | ns | >0.9999 |
|  |  |  |  |  |
| **Day 5** |  |  |  |  |
| WT vs. 5XFAD | -1.252 | -12.41 to 9.907 | ns | >0.9999 |
| WT vs. Tg4-42^hom^ | -12.36 | -24.06 to -0.6542 | * | 0.0323 |
| WT vs. 5XFAD/Tg4-42^hom^ | -18.09 | -31.09 to -5.094 | ** | 0.0016 |
| 5XFAD vs. Tg4-42^hom^ | -11.11 | -22.81 to 0.5979 | ns | 0.0733 |
| 5XFAD vs. 5XFAD/Tg4-42^hom^ | -16.84 | -29.84 to -3.842 | ** | 0.0041 |
| Tg4-42^hom^ vs. 5XFAD/Tg4-42^hom^ | -5.736 | -19.21 to 7.735 | ns | >0.9999 |

**Suppl. Table S3:** Within-day group comparison of latency in the MWM Acquisition trial at 6 months of age (2-way ANOVA with Bonferroni multiple comparisons test).


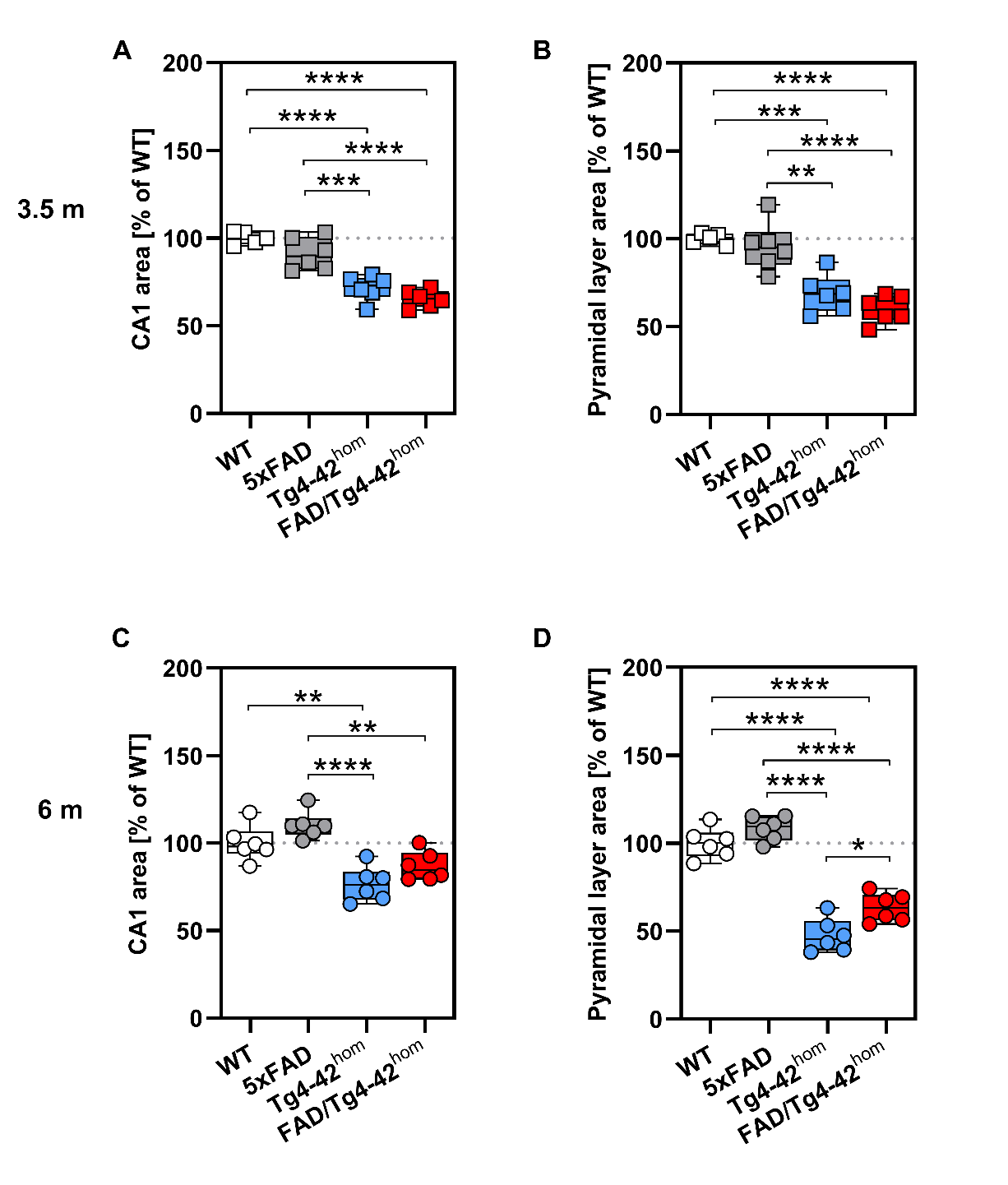


**Suppl. Fig. S6:** CA1 and pyramidal layer area measurements in 3.5- and 6-month-old animals. Young Tg4-42hom and 5XFAD/Tg4-42hom mice displayed a comparable reduction of CA1 and pyramidal layer areas (A,B). At 6 months of age, 5XFAD/Tg4-42^hom^ animals showed a significantly reduced shrinkage of the pyramidal layer area compared to age-matched Tg4-42^hom^ mice. One-way ANOVA followed by Bonferroni’s multiple comparison test. **p* < 0.05; ***p* < 0.01, ****p* < 0.001, *****p* < 0.0001.
